# A spatiotemporal multi-scale computational model for FDG PET imaging at different stages of tumor growth and angiogenesis

**DOI:** 10.1038/s41598-022-13345-4

**Published:** 2022-06-16

**Authors:** Farshad Moradi Kashkooli, Mohammad Amin Abazari, M. Soltani, Mehran Akbarpour Ghazani, Arman Rahmim

**Affiliations:** 1 Department of Mechanical Engineering, K. N. Toosi University of Technology, Tehran, Iran; 2 Department of Electrical and Computer Engineering, Faculty of Engineering, School of Optometry and Vision Science, Faculty of Science, University of Waterloo, Waterloo, Canada; 3 Advanced Bioengineering Initiative Center, Multidisciplinary International Complex, K. N. Toosi University of Technology, Tehran, Iran; 4 Centre for Biotechnology and Bioengineering (CBB), University of Waterloo, Waterloo, ON Canada; 5 Faculty of Mechanical Engineering, University of Tabriz, Tabriz, Iran; 6 Department of Integrative Oncology, BC Cancer Research Institute, Vancouver, BC Canada; 7 Department of Physics and Astronomy, University of British Columbia, Vancouver, BC Canada; 8 Department of Radiology, University of British Columbia, Vancouver, BC Canada

**Keywords:** Computational biophysics, Tumour angiogenesis, Tumour heterogeneity, Cancer imaging, Cancer models, Oncology, Biomedical engineering

## Abstract

A deeper understanding of the tumor microenvironment (TME) and its role in metabolic activity at different stages of vascularized tumors can provide useful insights into cancer progression and better support clinical assessments. In this study, a robust and comprehensive multi-scale computational model for spatiotemporal transport of F-18 fluorodeoxyglucose (FDG) is developed to incorporate important aspects of the TME, spanning subcellular-, cellular-, and tissue-level scales. Our mathematical model includes biophysiological details, such as radiopharmaceutical transport within interstitial space via convection and diffusion mechanisms, radiopharmaceutical exchange between intracellular and extracellular matrices by glucose transporters, cellular uptake of radiopharmaceutical, as well as its intracellular phosphorylation by the enzyme. Further, to examine the effects of tumor size by varying microvascular densities (MVDs) on FDG dynamics, four different capillary networks are generated by angiogenesis modeling. Results demonstrate that as tumor grows, its MVD increases, and hence, the spatiotemporal distribution of total FDG uptake by tumor tissue changes towards a more homogenous distribution. In addition, spatiotemporal distributions in tumor with lower MVD have relatively smaller magnitudes, due to the lower diffusion rate of FDG as well as lower local intravenous FDG release. Since mean standardized uptake value (SUV_mean_) differs at various stages of microvascular networks with different tumor sizes, it may be meaningful to normalize the measured values by tumor size and the MVD prior to routine clinical reporting. Overall, the present framework has the potential for more accurate investigation of biological phenomena within TME towards personalized medicine.

## Introduction

**Figure 1:**
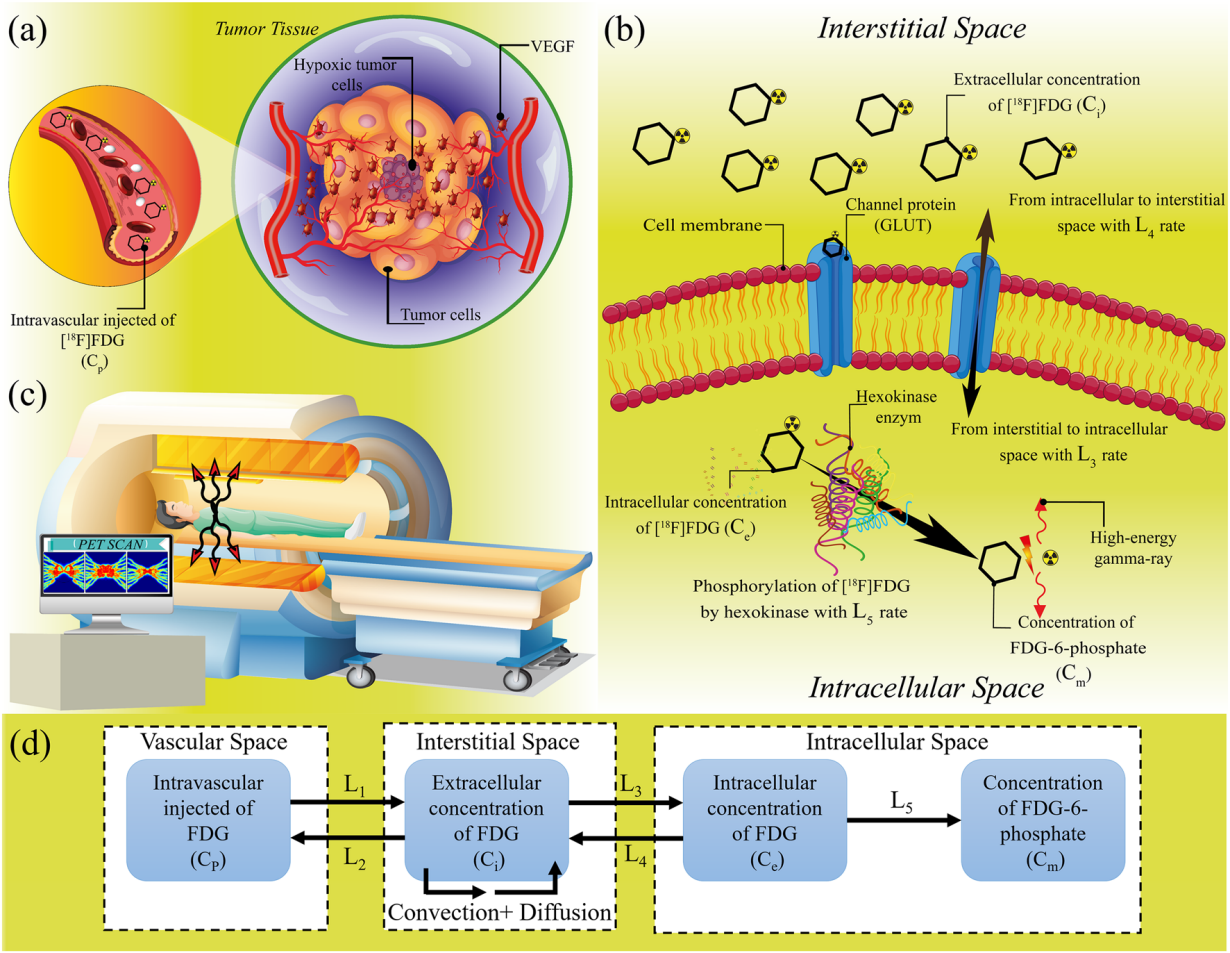
A schematic illustration of the present multi-scale computational model. The subcellular scale is the primary scale of the model, containing biochemical agents, including ECM (fibronectin gradient-induced haptotaxis for sprouting), matrix metalloproteinases, and VEGF gradient-induced chemotaxis. At the cellular scale, it consists of EC phenotypes, including tip cell migration. At the tissue level, this model also incorporates microvessel growth and remodeling, which is affected by mechanobiological and biochemical signals from wall shear stress with accurate hemodynamics and hemorheology. (**a**) Hypoxic tumor cells that have been deprived of oxygen, release some chemical agents (i.e., VEGF) which result in the formation of new microvessels from pre-existing vessels. Capillary networks induced by tumor angiogenesis play as a source for release of nutrients, therapeutic agents, as well as FDG molecules, which are injected into the patient’s bloodstream, (**b**) Extracellular FDG molecules can transport from tumor tissue to intracellular space and vice versa by GLUTs through L3 and L4 constant rate, respectively. Subsequently, each absorbed FDG molecule may phosphorylate by hexokinase enzymes to phosphorylated FDG via L5 constant rate. This process releases two high-energy gamma-rays in opposite directions, which can pass through the tissue, and (**c**) PET machine can detect these high-energy rays and hence via computer processing of the series of images taken in different angels the clinicians can detect the tumor tissue status for their further clinical decision-making process. (**d**) According to the FDG transport processes, a multi-compartment model is used in the spatiotemporal modeling of FDG transport. It should be mentioned that Microsoft Office PowerPoint 365 was used to create this figure.

Nuclear medicine imaging, particularly utilizing positron emission tomography (PET), is routinely used to assist oncologists in decision-making processes. Routine clinical applications of PET imaging include diagnosis and prognosis capabilities, initial staging, restaging, monitoring response to treatment, and predicting the risk of progression^1,2^. The PET scanner produces an image of the spatially varying concentrations of positron-emitting radioisotope-labeled pharmaceuticals. F-18 fluorodeoxyglucose (FDG) is one such radiopharmaceutical, used ubiquitously in the clinic; e.g., enabling more accurate detection of primary tumors as well as nodal and distant forms of metastatic disease^3^. As shown in Fig. 1, following intravenous injection, FDG molecules can transport into the extracellular matrix (ECM), where they may exchange between intracellular and extracellular matrices by glucose transporters (GLUTs)^1–3^. The effectiveness of FDG-PET imaging is related to its trapping in the glycolytic pathway after the early step of phosphorylation by the hexokinase enzyme, due to the negative charge of the added phosphate group. As such, the rate of FDG accumulation expresses information about the rate of glucose metabolism. Since cancer tumors generally depict higher glucose metabolism relevant to surrounding normal tissues^1,2^, the glucose analog FDG represents a valuable approach for cancer detection objectively and quantitatively.

Static and dynamic imaging are two most utilized techniques in PET-based molecular imaging for quantitative assessments of radioactivity concentrations over time across the regions of interest (ROIs). Static imaging (i.e., single time frame) includes acquiring snapshots of each PET bed position successively. In contrast, dynamic imaging includes obtaining a PET bed position continuously^2,4^ or simplified modeling of radiotracer kinetic, ranging from Patlak streamlined graphical modeling^5^ to full compartmental analysis^6^. Although PET kinetic modeling is a gold standard for absolute quantification of radio-labeled molecular probes^2^, it cannot incorporate spatiotemporal transport of radiopharmaceuticals by convection or diffusion processes between different compartments. In addition, these models may not fully account for certain individual underlying biophysiological features such as the conductivity of microvessels, permeability of transvascular and interstitial space variables^7,8^. Spatiotemporal distribution models (SDMs), which utilize systems of partial differential equations (PDEs) in contrast to ordinary differential equations (ODEs), are generally used in compartment models (i.e., pharmacokinetic modeling)^9–11^. They can also be used to investigate radiopharmaceutical redistributions over time and space simultaneously. As such, SDMs consider the effect of a range of physiological parameters within the tumor microenvironment (TME), including interstitial fluid fields, hydraulic conductivity, microvessel permeability, and microvascular structures.

The potential of SDMs in drug delivery systems has been widely demonstrated, as they enable measuring the solute transport by convection, diffusion, and reaction mechanisms^12–15^. Meanwhile, only a few studies have investigated the spatiotemporal distribution of PET radiotracer in solid tumors^7,8,16–19,73^. Some studies have focused on the spatiotemporal distribution of hypoxia-PET radiotracers in which the effects of interstitial fluid fields and lymphatic drainage have not been considered^8,16,17^. Some have presented microscopic-scale methods incorporating only radial molecular diffusion^16,17^ or employing simple models of vessel architectures^18^. In all studies mentioned, convection and diffusion transport mechanisms from vessel to tissue or within the tissue were not considered, which may influence the modeling of radiopharmaceutical distributions in the ROIs. Moreover, a multi-scale model to study the spatiotemporal distribution of radiopharmaceuticals at different stages of tumor progression with various tumor sizes, as well as its individual microvascular structure, is of great importance. Such a model can help the clinicians improve their understanding of the relation between the distributions of accumulated FGD radiotracer at different stages of angiogenesis with varying microvascular densities (MVDs).

Tumor vasculature and angiogenesis process have critical roles in the response of solid tumors to systemic therapies, greatly affecting the FDG transport. Tumor-induced angiogenesis is a multi-scale process that consists of the formation of new vessels from pre-existing vasculatures to pave the way to further tumor growth^20^. In detail, in response to starving central hypoxic tumor cells, due to depletion of surrounding cells, several angiogenic and chemotactic growth factors_e.g., vascular endothelial growth factor (VEGF)_are secreted^21^. Subsequently, as shown in Fig. 1a, the initial stage of angiogenesis is created by VEGF binding to its receptor on tip endothelial cells (ECs) that extended from the existing vessel wall and migration of vessel sprouts towards areas within the TME with higher angiogenic factors (i.e., chemotaxis) and fibronectin (i.e., haptotaxis) gradients^22,23^. In the following, by distancing capillary sprouts from their parent vessel, they form several loops and branching, and finally, create a complex network of new capillaries that supports blood flow into the tumor^24^.

In the present work, a robust, comprehensive, and multi-scale computational model is developed to investigate the spatiotemporal distribution of FDG at different stages of tumor angiogenesis. As shown in Fig. 1, this model includes subcellular-, cellular-, and tissue-level scales. Present SDM also considers lymphatic systems explicitly and calculates the interstitial and intravascular fluid flow at the interstitium and across the microvascular networks, which influences the extracellular and intracellular distribution of radiopharmaceuticals. Further, the effects of different microvascular networks (with various MVDs) and tumor sizes on the association and disassociation of FDG to glucose receptors, FDG internalization to cancer cells, as well as intracellular phosphorylated FDG by enzyme have also been considered. Unlike many previous studies that considered the concentration of FDG as the only factor determining metabolic activity, the present model includes multiple standardized uptake value (SUV)-based features to determine cellular activity and characterize tumor FDG uptake. Ultimately, different concentrations of FDG, time-activity curves (TACs), as well as SUV metrics in each tumor stage are calculated and compared with each other. Results of the present model are validated by several previously published in vivo and in silico experiments. Analyzing personalized biological features has the potential of providing a more comprehensive framework, which can improve disease assessment in individual cancer patients and uptake prediction in radiopharmaceutical therapies, treatment prediction, and patient outcomes.

## Materials and methods

Transport of FDG in the interstitium and intracellular spaces includes the following mechanisms, as demonstrated in Fig. 1:

1. Fluid flow and mass transport within a microvascular network.
2. Mass and momentum conservation for interstitial fluid flow.
3. Mass transport for the extracellular, intracellular, and phosphorylated intracellular tracer concentrations.

In the present section, first, detailed mathematical modeling is provided, including equations governing the angiogenesis modeling, interstitial fluid flow, and FDG transport. Then, the computational domain, and boundary conditions are presented. Finally, the solution strategy, the grid independence test, and model validation are discussed.

### Tumor angiogenesis modeling

In the current study, a dynamic adaptive microvascular network modeling is used to simulate capillary networks induced by tumor angiogenesis. Present model is based on a discrete probabilistic model, which was developed by Anderson et al.^21^ and Soltani et al.^13^. This model considers branching, anastomosis, blood flow, hematocrit, wall shear stress, and consequently blood flow induced vessel branching. It is assumed that filopodias situated on the tip ECs route the trajectories and direct tip ECs, while stalk ECs proliferate and elongate the vessel. EC movement toward the tumor is affected by three mechanisms^25^: (1) random movement, (2) direct movement due to VEGF gradients secreted by hypoxic tumor cells (i.e., chemotaxis), and (3) transverse movement due to gradient of insoluble chemicals (i.e., haptotaxis). A lattice-based grid generation has been used to decrease both time and cost of simulations. Therefore, an ensemble of cells moves together as the simulation marches in time. Posterior to vasculature generation, laminar blood flows across the networks as it is a requisite to blood vessel perseverance^26^. By flowing blood through the microvessels, vessels may dilate or constrict consecutively as the dependence of blood properties and wall shear stress are reciprocal, based on the theory of blood flow-induced branching, the vessel segments with high shear rates and high concentrations of VEGF form new sprouts and branch^27,28,73^. A detailed description of the mathematical model of angiogenesis, hemodynamics and interstitial fluid flow, hemorheology, and dynamic structure adaptation method are presented in Supplementary File.

### Fluid flow in interstitium

The momentum and continuity equations in the interstitium space are solved to provide the velocity and pressure. The momentum equation in biological tissues, as porous media, is simplified to Darcy’s law^12^, which illustrates the relationship between interstitial fluid velocity (IFV) and interstitial fluid pressure (IFP) as^12,13,29^:

1 $${\overrightarrow{V}}_{i}=-\kappa \nabla {P}_{i},$$

where ${\overrightarrow{V}}_{i}$, ${P}_{i}$, and $\kappa$ are IFV, IFP, and hydraulic conductivity of interstitium, respectively.

The continuity equation in the biological tissues, by considering the presence of source/sink terms, is modified as^12,13,29^:

2 $$\nabla .{\overrightarrow{V}}_{i}={\phi }_{v}-{\phi }_{l},$$

in which ${\phi }_{v}$ is the net fluid flow per volume from microvessels to the interstitium and vice versa, and ${\phi }_{l}$ is the net fluid flow per volume from interstitium to lymphatic microvessels, which are described by Starling’s law as^12,13,29^:

3 $${\phi }_{v}={L}_{P}\frac{S}{V}\left({P}_{B}-{P}_{i}-{\sigma }_{s}\left({\pi }_{B}-{\pi }_{i}\right)\right),$$

4 $${\phi }_{l}={L}_{PL}{\left(\frac{S}{V}\right)}_{L}\left({P}_{i}-{P}_{L}\right),$$

where ${P}_{B}$ is intravascular blood pressure, and ${\pi }_{B}$ plasma oncotic pressure, ${\pi }_{i}$, interstitial fluid oncotic pressure, and ${P}_{L}$ the hydrostatic pressure of the lymphatic vessel. The detailed definition of related parameters with their values is provided in Table 1.

**Table 1:** Parameters of interstitial transport used in numerical simulations.

Parameter	Definition	Value (tissue type)	Unit	References
${\pi }_{B}$	Oncotic pressure of microvessels	20 (healthy) 20 (tumor)	mmHg	^7,73^
${\pi }_{i}$	Oncotic pressure of interstitial fluid	10 (healthy) 15 (tumor)	mmHg	^7,73^
${\sigma }_{s}$	Coefficient of average osmotic reflection	0.91 (healthy) 0.82 (tumor)	–	^7,73^
${L}_{P}$	Hydraulic conductivity of the microvessel wall	0.27 × 10^–11^ (healthy) 2.1 × 10^–11^ (tumor)	m/(Pa s)	^7,73^
${L}_{PL}{\left(\frac{S}{V}\right)}_{L}$	Coefficient of lymph filtration	1 × 10^–7^ (healthy)	1/(Pa s)	^7,73^
$\kappa$	Hydraulic conductivity of interstitium	8.53 × 10^–9^ (healthy) 4.13 × 10^–8^ (tumor)	cm^2^/(mmHg s)	^7,73^
${P}_{L}$	Hydrostatic pressure of lymph vessels	0 (healthy)	Pa	^7,73^

### Modeling of radiopharmaceutical transport

In the current study, radiopharmaceutical transport in the SDM is described by the convection–diffusion–reaction (CDR) equations with the aim of obtaining a more patient-specific related model. This system of equations is employed for the radiopharmaceutical transport between different compartments via diffusion and convection mechanisms. They potentially investigate several biochemical and physiological phenomena, including radiopharmaceutical transport across the microvessels due to diffusion and convection process, transport rate in interstitial space by diffusion and convection in tissue, as well as cell binding and uptake. Therefore, based on the processes of FDG transport in different compartments, as demonstrated in Fig. 1, the system of equations of the SDM is represented as follows^30,73^:

5 $$\frac{\partial {C}_{i}}{\partial t}={-{\overrightarrow{V}}_{i}\nabla {C}_{i}+D}_{eff}{\nabla }^{2}{C}_{i}-{L}_{3}{C}_{i}+{L}_{4}{C}_{e}+{\Phi }_{V}-{\Phi }_{L},$$

6 $$\frac{\partial {C}_{e}}{\partial t}={L}_{3}{C}_{i}-{L}_{4}{C}_{e}-{L}_{5}{C}_{e},$$

7 $$\frac{\partial {C}_{m}}{\partial t}={L}_{5}{C}_{e},$$

8 $${L}_{1}={\phi }_{v}\left(1-{\sigma }_{f}\right)-\frac{{P}_{m}S}{V}\frac{Pe}{\mathrm{exp}\left(Pe\right)-1},$$

9 $${L}_{2}=\frac{{P}_{m}S}{V}\frac{Pe}{\mathrm{exp}\left(Pe\right)-1}+{\phi }_{l},$$

10 $$Pe=\frac{{\phi }_{v}(1-{\sigma }_{f})}{{P}_{m}S/V},$$

where ${C}_{i}$ is the extracellular concentration of FDG normalized to extracellular volume, ${C}_{e}$ is FDG intracellular concentration, and ${C}_{m}$ is phosphorylated intracellular (FDG-6-P) concentration. ${L}_{1}$, ${L}_{2}$ are the exchange rate parameters between plasma and ECM and ${L}_{3}$, ${L}_{4}$, and ${L}_{5}$ are defined as the transport rate constant between the ECM and tumor cell, the inverse rate from tumor cell to the ECM, and the phosphorylation rate of FDG, respectively. ${D}_{eff}$ is the effective diffusion coefficient, $Pe$ is the Peclet number, ${\sigma }_{f}$ is the filtration reflection coefficient and ${P}_{m}$ is the permeability coefficient of microvessels.

${\Phi }_{V}$ and ${\Phi }_{L}$ represent radiopharmaceutical transport rate per unit volume from microvessels into the interstitial space and from the interstitial space into the lymphatic microvessels, respectively. ${\Phi }_{V}$ and ${\Phi }_{L}$ are defined based on Patlak’s model, as^12,31,73^:

11 $${\Phi }_{V}={\phi }_{v}\left(1-{\sigma }_{f}\right){C}_{p}+{P}_{m}\frac{S}{V}\left({C}_{p}-{C}_{i}\right)\frac{Pe}{\mathrm{exp}\left(Pe\right)-1},$$

12 $${\Phi }_{L}={\phi }_{l}{C}_{i},$$

in which ${C}_{p}$ demonstrates FDG plasma arterial concentration. Related parameters in radiopharmaceutical transport modeling are defined in Table 2.

**Table 2:** Parameters for spatiotemporal distribution modeling of radiopharmaceutical transport.

Parameter	Definition	Value (tissue type)	Unit	References
${D}_{eff}$	Effective diffusion coefficient	0.37 × 10^–9^ (healthy) 1.23 × 10^–9^ (tumor)	m^2^/s	^32,33,73^
${P}_{m}$	Microvessel permeability coefficient	2.26 × 10^–6^ (healthy) 7.83 × 10^–6^ (tumor)	m/s	^8,73^
${\sigma }_{f}$	Filtration reflection coefficient	0.9	–	^7,73^
${L}_{3}$	Transport rate parameter into the cell	8.2 × 10^–4^	1/s	^34,73^
${L}_{4}$	Transport rate parameter out of the cell	6.7 × 10^–4^	1/s	^34,73^
${L}_{5}$	Phosphorylation rate	5.3 × 10^–4^	1/s	^34,73^

### Semi-quantitative assessment of FDG transport modeling

For quantitative analysis of radiopharmaceutical uptake at different stages of tumor progression, SUV is calculated. SUV is determined by the ratio of the total tissue radioactivity concentration investigated in an ROI to the radioactivity injected in the body, normalized by the body weight as shown^35^:

13 $$\mathrm{SUV}=\frac{\mathrm{Tissue \; radioactivity \; concentration }\left({C}_{total}\right)}{\mathrm{Injected \; radioactivity}}\times \mathrm{Body \; weight},$$

14 $${C}_{total}={C}_{i}+{C}_{e}+{C}_{m},$$

in which a patient with 75 kg weight is considered for further analysis^36^. It is worth noting that total FDG concentration (${C}_{total}$) is determined as a sum of three other concentrations (${C}_{i}$, ${C}_{e}$, and ${C}_{m}$).

### Computational domain and boundary condition

A 2D computational domain with the dimension of 5 × 5 cm^2^ is taken into consideration in which a solid tumor with a diameter D_Tumor_ is located at the center of its surrounding healthy tissue (Supplementary Fig. S1). Additionally, the capillary network grows from two parent vessels on the vertical lines of both sides of the computational domain to achieve a more realistic tumor model. To represent various stages of tumor progression, different tumor sizes—1 cm, 1.5 cm, 2 cm, 2.5 cm—with unique structures of microvessels for each size are considered. These diameter threshold values are chosen based on the previous works so that the tumor-induced angiogenesis process has been initiated^14,37,38^.

For intravascular blood flow simulation, the inlet and outlet pressure values of both parent vessels are selected based on the physiological-accurate boundary conditions determined in the literature^7,39,40^ as follows (Supplementary Fig. S1):

$${P}_{B, Inlet}=35 \left(\mathrm{mmHg}\right) \& \, {P}_{B, Outlet}=10 \left(\mathrm{mmHg}\right).$$

Continuity boundary conditions for interstitial flow fields and radiopharmaceutical concentration as well as its flux are selected at the inner boundary (i.e., the boundary between tumor and healthy tissue). Different boundary conditions used in the interstitial fluid flow and FDG concentration modeling are outlined in Table 3; where ${\Omega }^{n}$ and ${\Omega }^{t}$ denote the healthy and tumor tissues at their interface, respectively. Since the IFP at the outer boundary (i.e., boundary around healthy tissue) is fixed, a Dirichlet boundary condition is selected^13^. For radiopharmaceutical concentration at the outer boundary, an open-type boundary condition is used^7^. In addition, for radiopharmaceutical distribution modeling, a plasma TAC is obtained from Backes et al.^7,36^.

**Table 3:** Boundary conditions employed in computational modeling.

Region	Boundary conditions	
	Interstitial fluid flow	Radiopharmaceutical concentration
Inner boundary	$\left({-{\kappa }_{t}P}_{i}|{ }_{{\Omega }^{t}}\right)=\left({-{\kappa }_{n}P}_{i}|{ }_{{\Omega }^{n}}\right)$ $\left({P}_{i}|{ }_{{\Omega }^{t}}\right)=\left({P}_{i}|{ }_{{\Omega }^{n}}\right)$	$\left({{(D}_{eff}}^{t}\nabla C-{V}_{i}C)C|{ }_{{\Omega }^{t}}\right)=\left(({{D}_{eff}}^{n}\nabla C-{V}_{i}C)C|{ }_{{\Omega }^{n}}\right)$ $\left(C|{ }_{{\Omega }^{t}}\right)=\left(C|{ }_{{\Omega }^{n}}\right)$
Outer boundary	${P}_{i}=\mathrm{Constant}$	$-\mathrm{n}.\nabla C=0$

### Solution strategy

**Figure 2:**
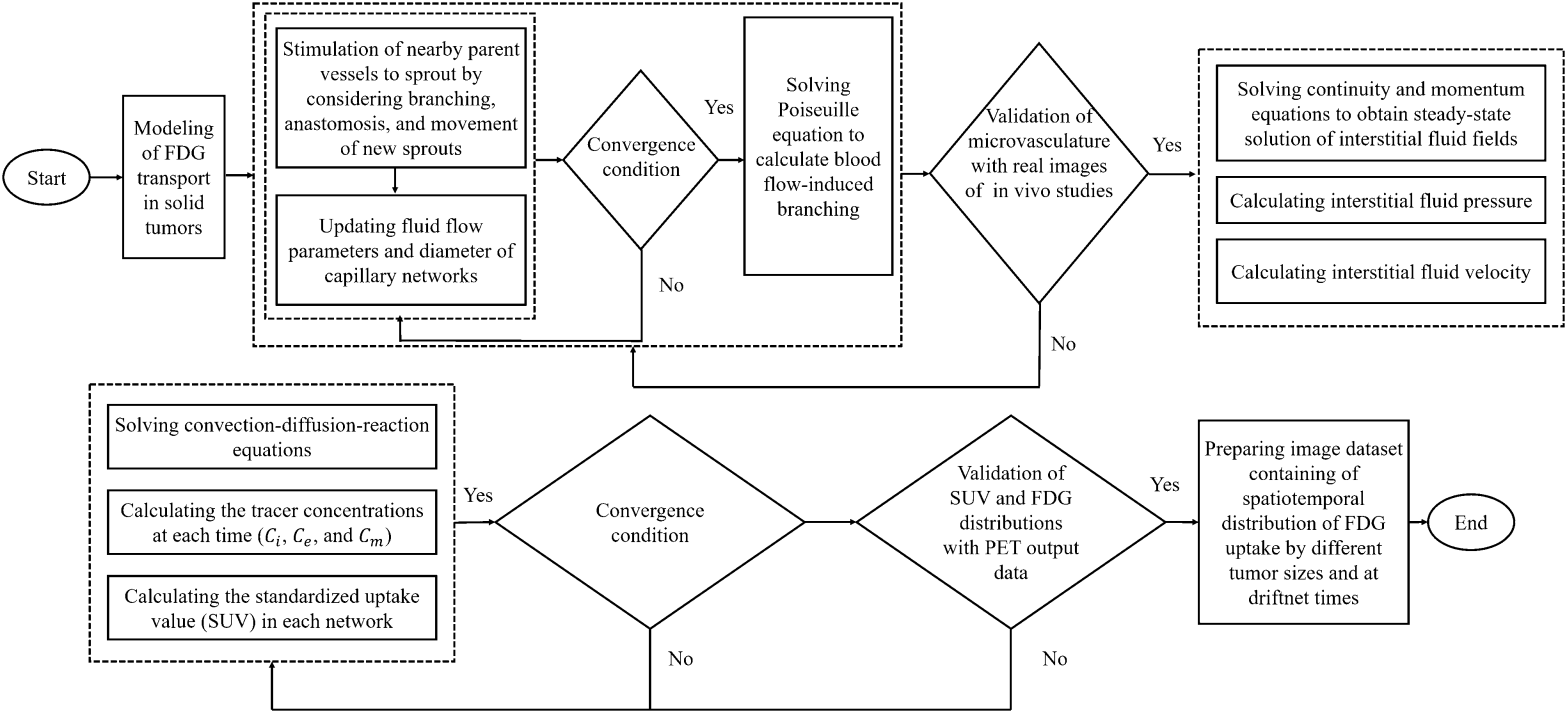
Flowchart used to step-by-step description of radiopharmaceutical transport modeling in solid tumors.

A step-by-step description of the methodology employed in the present study is shown in Fig. 2. First, microvascular networks during tumor‐induced angiogenesis are generated based on the different stages of tumor growth with various tumor diameters. Subsequently, the mass and momentum equations in the interstitium are solved stationary, after which the architecture of the microvascular network is validated qualitatively. IFP and IFV values are then utilized to solve the CDR equations. Subsequently, SUV is calculated in each stage of tumor progression based on different measured FDG concentrations. Ultimately, the distribution of SUV-based parameters in TME is quantitatively and qualitatively verified to be used in further investigations.

The governing equations, including continuity, Darcy, and CDR equations are solved using the commercial computational fluid dynamics (CFD) software COMSOL Multiphysics 5.5 (COMSOL, Inc., Burlington, MA, USA) which works based on the finite element method. The residual square errors are set to 6 orders of magnitudes. The grid independency test is also performed and the results of IFP and concentration of the FDG radiopharmaceutical for four different generated computational grids (i.e., coarse, medium, fine, and extremely fine) are evaluated. There is a maximum of about 3% difference between the medium and fine meshes, and about 1% variation between the fine and extremely fine grids. To save computational costs, the fine mesh with about 27,500 triangular elements is selected and used in the subsequent different simulations.

### Model validation

**Figure 3:**
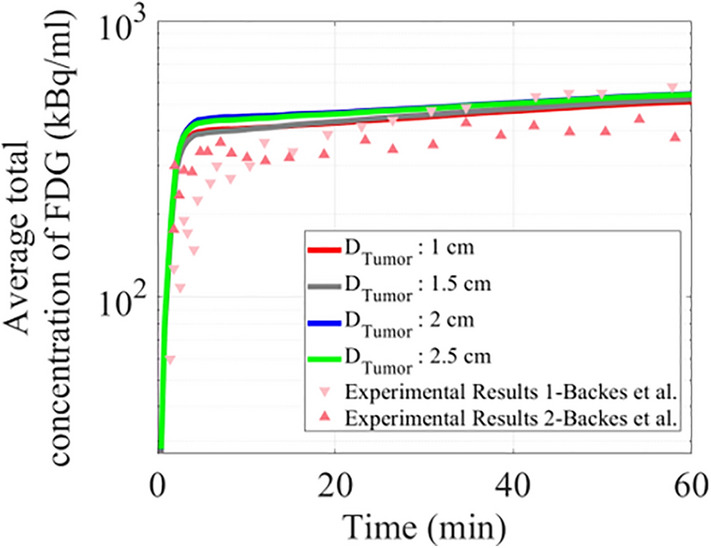
Comparison of simulated average FDG radiopharmaceutical uptake by tumors in different sizes and the experimental results of Backes et al.^36^.

Figure 3 depicts the comparison of the average FDG concentration in the tumor region, showing a remarkable correspondence between two experimental results of Backes et al.^36^ and the current numerical results by varying tumor sizes. Backes and co-authors determine reference tissue kinetic parameters in 11 rats from PET data and calculate the tissue TACs in rats suffering from acute focal cerebral ischemia. Despite the difference in boundary conditions and computational domains, the average FDG concentration uptake by solid tumors illustrates a similar trend compared to the experimental data. Given the complexity of the biology, physiology, as well as oncology under measurement, the exceptional arrangement obtained is evidence of the level of sophistication. As demonstrated, 20-min post-injection of FDG tracer, the total uptake in both extracellular and intracellular spaces has been matched with the first experimental data. Moreover, results of total average FDG uptake during tumor growth have the same trend compared to experimental studies published in the literature^34,41^ and our previous numerical studuies^7,73^. It should be noted that interstitial fluid fields, intravascular pressure values, the formation of microvessels, and the distribution of FDG uptake by tumor cells will be verified quantitatively and qualitatively with the literature in the following sections.

## Results

In the present study, four different complex vascular networks during tumor‐induced angiogenesis are presented. To obtain the spatial and temporal distribution of FDG concentration, Darcy and continuity equations in the interstitium, as well as CDR equations are solved simultaneously. In the following, the distribution of IFP, IFV, intravascular pressure, FDG concentration, and related SUV-based parameters in the tumor and healthy tissues will be evaluated in-depth.

### Intravascular and interstitial fluid flow

**Figure 4:**
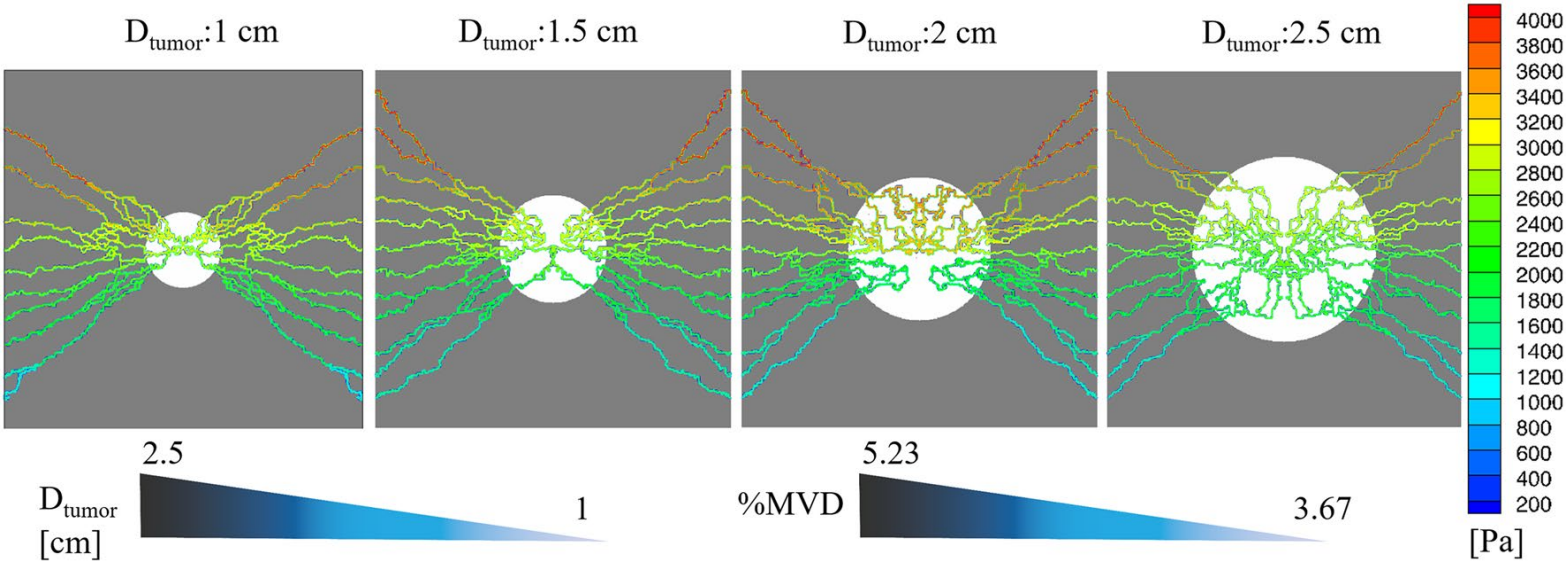
Intravascular pressure distribution and %MVD at different stages of vascularized tumor progression. MVD is increased as the tumor diameter increases (white and gray color regions indicate tumor and healthy tissues, respectively).

Tumor-induced vascular networks with their pressure values for tumor sizes of 1–2.5 cm are shown in Fig. 4. In addition, the growth of microvessels from parent vessels in tumors with 1 and 2 cm diameters is provided in Supplementary File (Supplementary Figs. S2 and S3, respectively). The percentages of MVD (%MVD) for each capillary network are calculated by dividing the microvessel nods by all computational domain nodes (see Supplementary Information, numerical method section). MVD for 1–2.5 cm tumor sizes is calculated as 3.67, 4.25, 4.72, 5.23%, respectively. In other words, by increasing the tumor size, the heterogeneous distribution of tumor-associated vasculature is also increased. Intravascular pressure in all tumor sizes has almost similar values (an average of 2660–3325 Pa).

**Figure 5:**
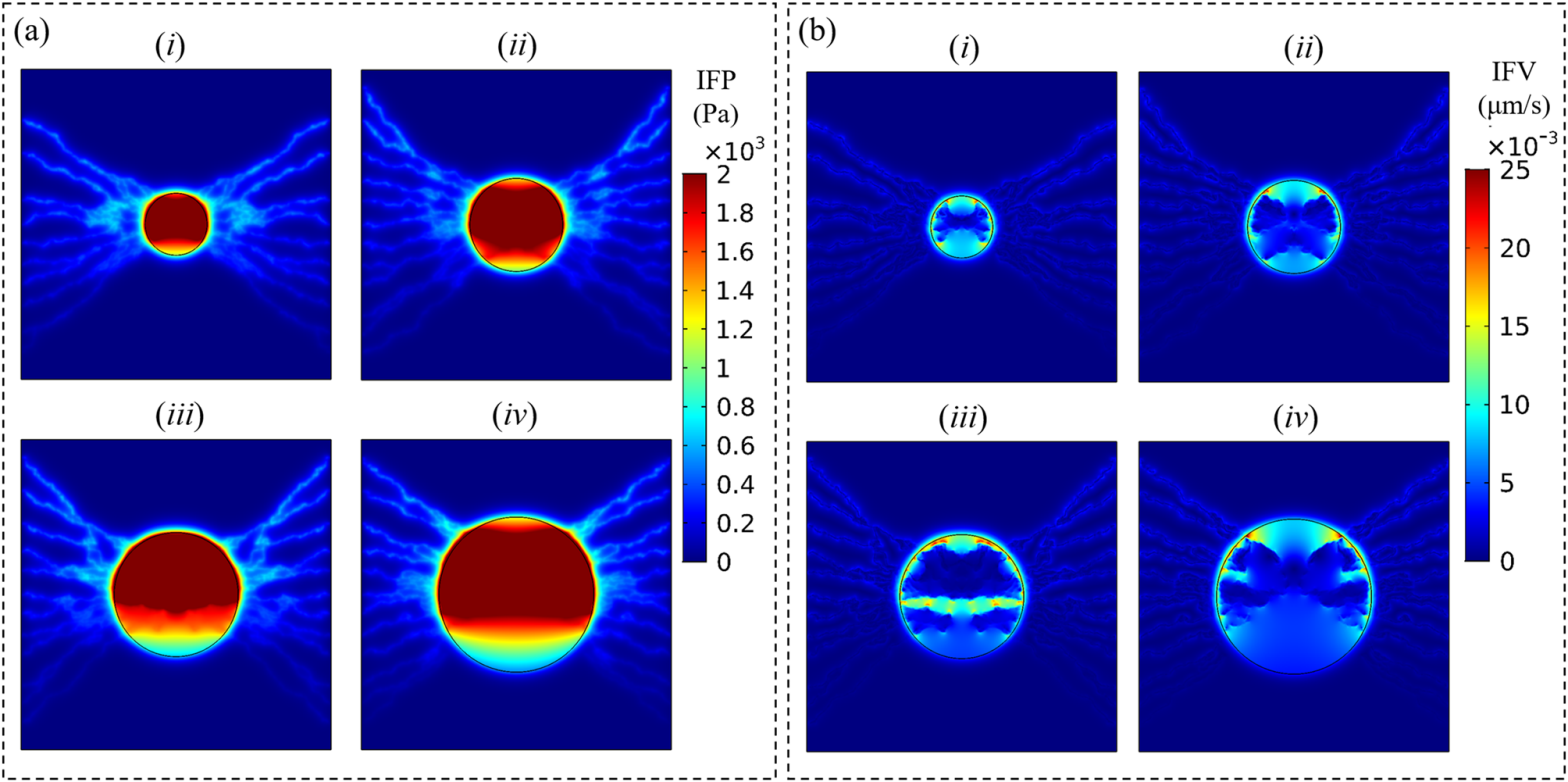
Interstitial fluid fields. (**a**) IFP and (**b**) IFV for tumors with (*i*) 1 cm, (*ii*) 1.5 cm, (*iii*) 2 cm, and (*iv*) 2.5 cm diameter.

Two major components of the biomechanical microenvironment of cancer are the IFP and IFV, which are illustrated in Fig. 5a,b, respectively. Maximum IFP in all stages of tumor progression is seen within the tumor tissue. The average of maximum IFP in the tumor regions is about 2.94 kPa. In other words, the maximum IFP value increases slightly from 2.7 kPa in 1-cm tumor to 3.1 and 3.2 kPa in tumor sizes of 2 and 2.5 cm, respectively. IFP distribution does not vary significantly between various networks during tumor growth and only a few variations may be observed. The IFP has a spatially lower value at the bottom and upper regions of tumor tissue in each network, where the MVD is lower therein. Figure 5b indicates the non-uniform distribution of IFV in TME. IFV has a low value, less than 3 × 10^–8^ m/s, throughout the whole domain, unless at the tumor border (i.e., the interface of tumor and healthy tissues), where reaches its maximum value.

### Radiopharmaceutical concentration

Distribution of different FDG concentrations at various time intervals post-injection is presented in Supplementary Fig. S3. For a better comparison of the results, FDG concentrations have been non-dimensionalized by dividing each one by a constant value (750 kBq/ml, which is the average maximal value in the domain). At the initial moments of radiotracer injection, FDG is located only within the microvessels but they are rapidly extravasated across the wall of microvessels through the convection and diffusion mechanisms. The extracellular FDG concentration (${C}_{i}$) is prevailing at early time steps and it is quickly transported into intracellular space (${C}_{e}$). Intracellular FDG gradually turns into phosphorylated intracellular FDG (${C}_{m}$) over time and finally accumulates intracellularly. FDG concentration within cancerous tissue in all tumor sizes is several times higher than the concentration in healthy tissue.

**Figure 6:**
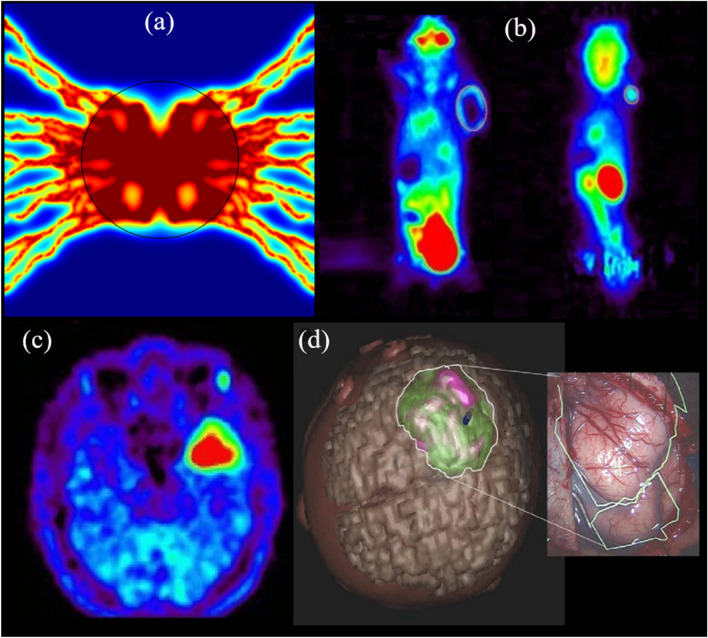
Qualitative validation of present computational results with longitudinal FDG-PET images of two tumor-bearing mice and a pediatric brain tumor. (**a**) Synthetic PET image that is generated by the current mathematical model. (**b**) Longitudinal FDG-microPET scans of mice with glioblastoma U87 (right picture) and adenocarcinoma MDA-MB-231 (left picture) tumor cells^42^. The tumors can be easily detectable on micro-PET images by red color regions. (**c**) Preoperative PET image in a 10-month-old boy with parietal ganglioglioma^43^. (**d**) Tumor contours with its vessels are illustrated in the preoperative 3D planning. Reprinted by permission from Refs.^42,43^.

A qualitative comparison between the present synthetic images of PET produced by mathematical modeling and the real FDG-PET images in two tumor-bearing mice as well as in a 10-month-old boy with parietal ganglioglioma is illustrated in Fig. 6. Present computational results demonstrate the higher FDG uptake in tumor tissue compared to healthy tissue, which is consistent with in vivo observations of Sha et al.^42^ and Pirotte et al.^43^. In agreement with in vivo observations, created color gradient demonstrates that tumor tissue can be characterized by higher FDG uptake compared to healthy tissues.

**Figure 7:**
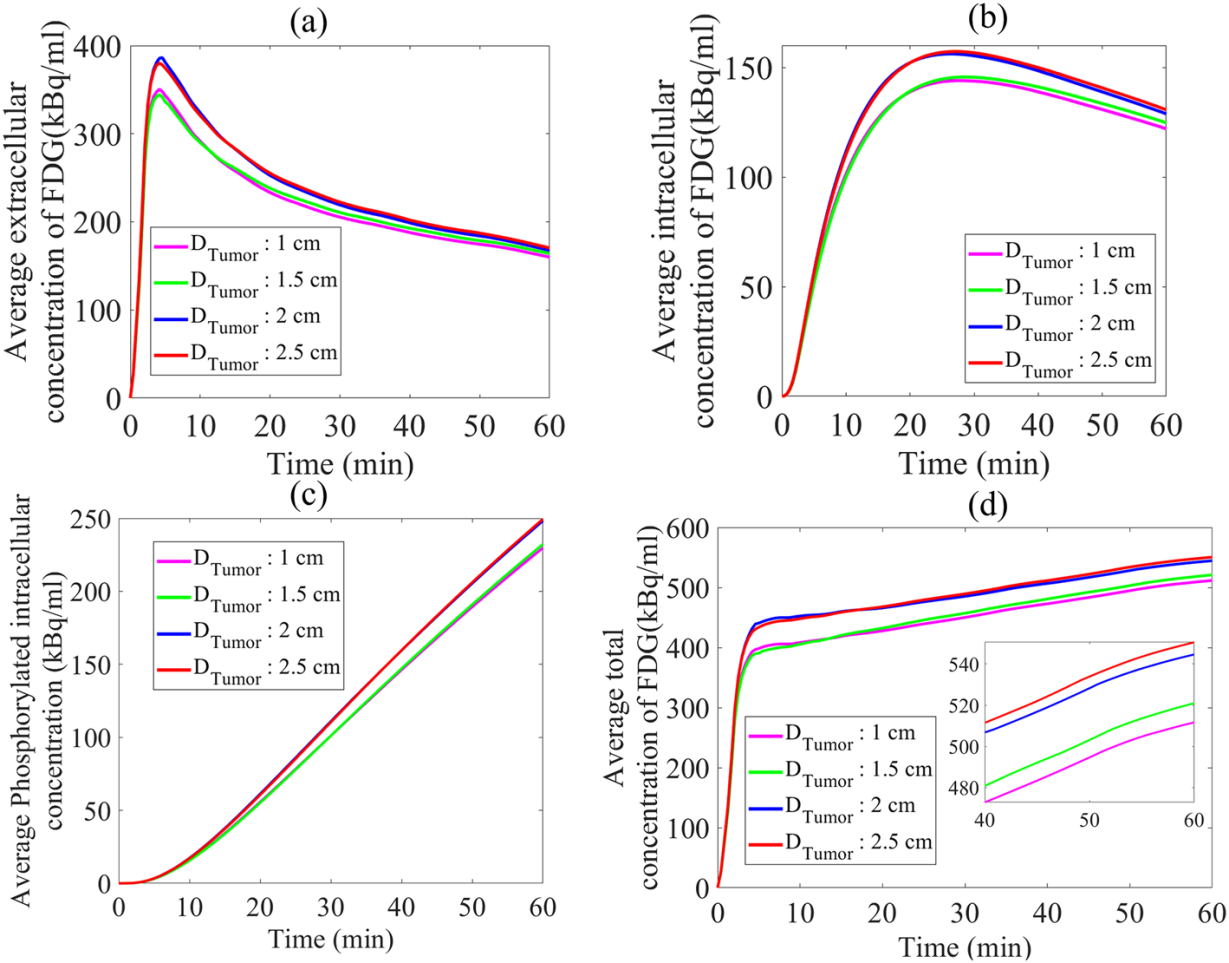
TACs of different concentrations of FDG. (**a**) Extracellular FDG, (**b**) intracellular FDG, (**c**) phosphorylated intracellular FDG, and (**d**) total FDG concentration within the tumor tissue.

TACs of different FDG concentrations across the tumor tissue for different sizes are shown in Fig. 7. During the first five minutes of injection, extracellular FDG concentration in all tumor sizes has increased until reaching its maximum value and then gradually transported into the cancer cells. Results of total FDG uptake indicate that tumors with 2.5 and 1.5 cm diameters absorb about 8% and 2% higher FDG compared to 1 cm tumor, respectively. Overall, one-hour post-injection, for all the concentration results, the tumor with a larger diameter has relatively shown a higher amount of FDG uptake.

### Semi-quantitative measurement of radiopharmaceutical transport modeling

**Figure 8:**
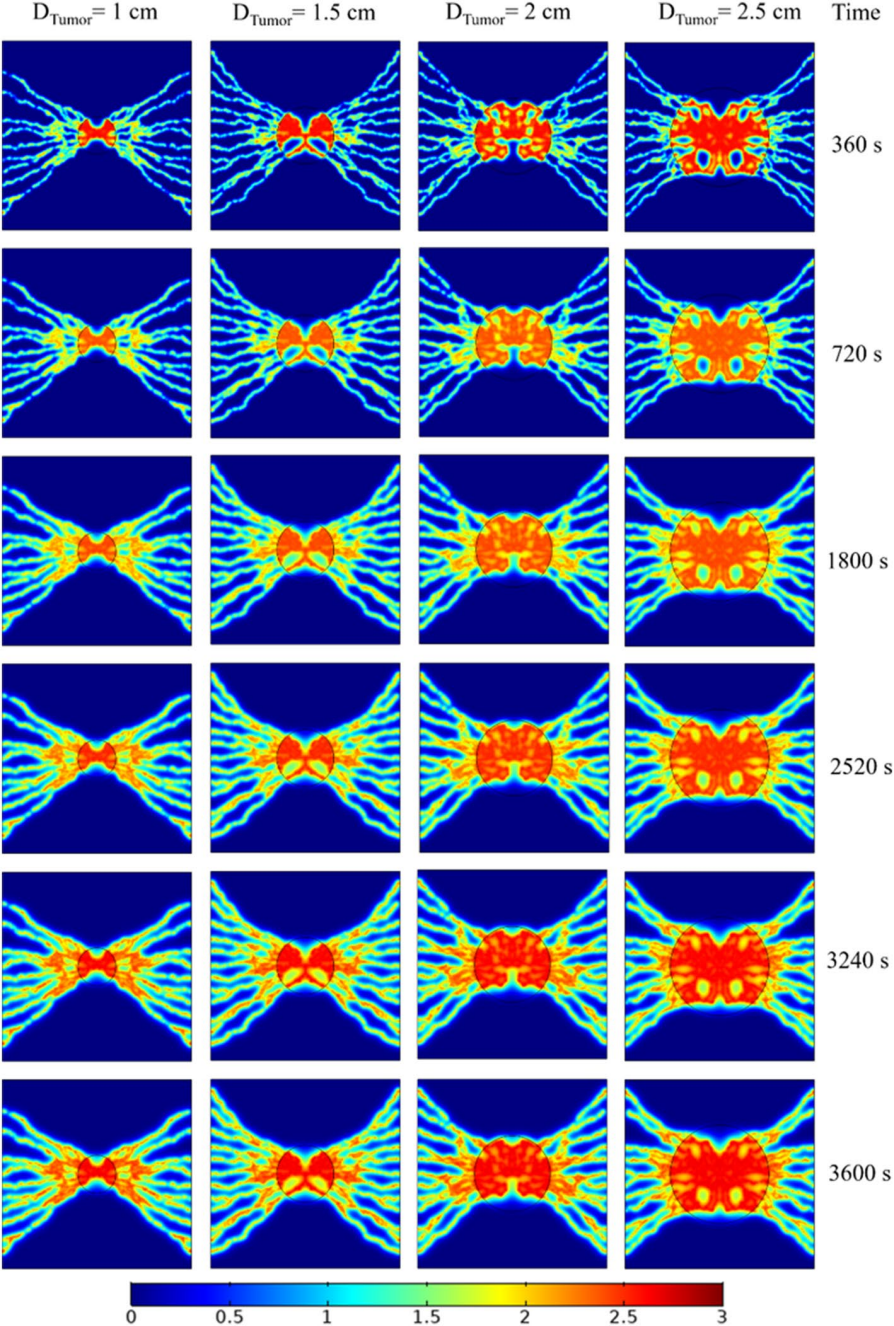
Spatiotemporal distribution of SUV at various times post FDG injection for different tumor sizes.

In tumor imaging using PET, SUV is the commonly used indicator for characterizing tumor uptake. Indeed, SUV thresholds are utilized to distinguish malignant from benign disorders. Figure 8 provides an overview of the spatiotemporal distribution of SUVs in different tumor sizes investigated in the present work. Moreover, Supplementary Videos 1 and 2 demonstrate how the SUV was distributed within 1 and 2 cm tumor sizes, respectively. For all examined tumor sizes, at initial times post-injection, a high concentration of FDG at the tumor core diffuses throughout the TME into the regions with the lower MVD. Similar to total FDG concentration distribution, SUV has its maximum value within the tumor tissue in all tumor sizes at different time frames. Results illustrate that larger tumors have higher SUVs, in which the SUV in healthy tissue has approximately a median maximal value of 0.65. In tumor tissues, the maximum SUV reaches 2.75.

**Figure 9:**
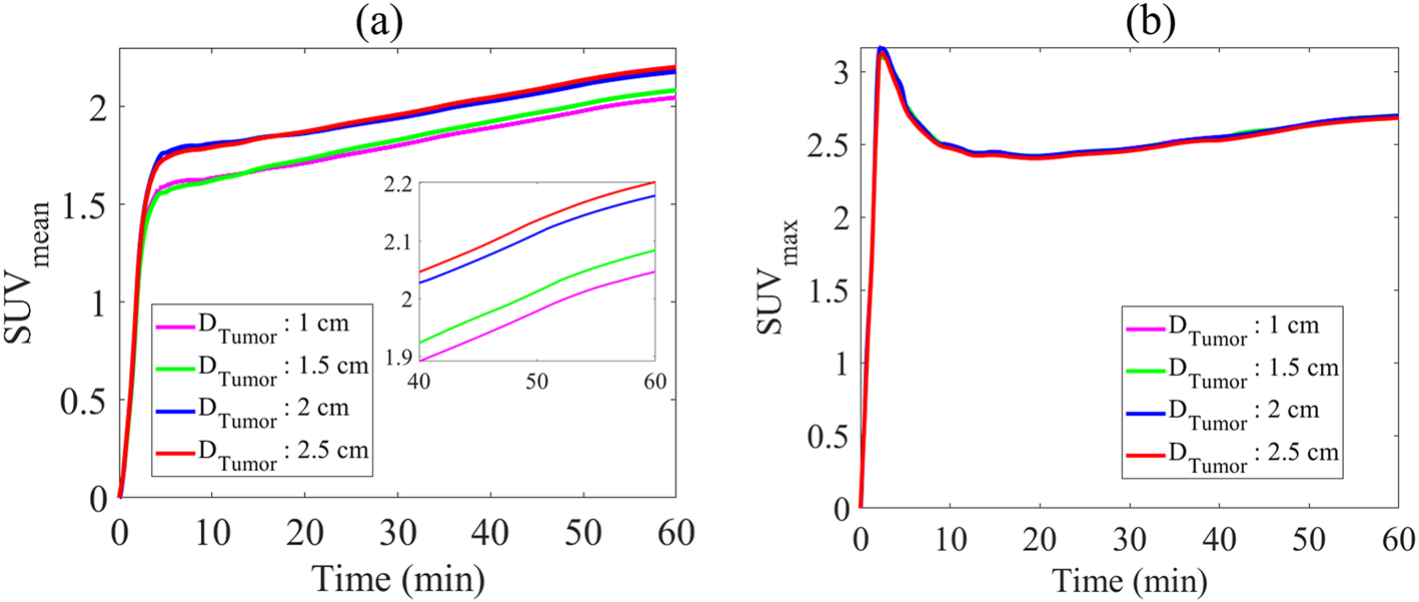
Time-radioactivity curves of different tumor sizes. (**a**) Mean and (**b**) maximum SUV across the tumor tissue.

SUV_mean_, the mean pixel activity evaluated in the ROI, and SUV_max_, the maximum pixel activity evaluated in the ROI, are two meaningful metrics widely used in clinical-decision support by radiologists and oncologists. TACs of these two parameters within tumor tissue in four examined tumor sizes are illustrated in Fig. 9. SUV_mean_, like total FDG concentration (${C}_{total}$), increases over time, where larger tumors have relatively higher SUV_mean_. According to Fig. 8b, SUV_max_ increases over time, but no large significant correlation is found between SUV_max_ and different tumor stages.

## Discussion

According to Fig. 4, tumor growth greatly affects the microvessels’ distribution within the ECM, especially inside the tumor tissue, where the gradient of VEGF is much higher than that of healthy tissue. Results of the formation of capillary networks show an excellent qualitative agreement with the previous in vivo experiments^44,45^ and a more recent study by Rebling et al.^46^, who proposed a non-invasive and label-free imaging framework for long-term visualization of blood microvessels in non-injured dorsal mouse skin. In a general way, the transverse and longitudinal movement of tumor-induced microvascular networks are increased by tumor growth, indicating enhanced angiogenesis rate and higher heterogeneous distribution of microvessels over TME. In other words, by increasing the tumor size, a greater proportion of the tumor area is covered by microvessels. Moreover, average intravascular pressure values in all tumor sizes are in good correspondence with the numerical studies^7,13,73^ and physiological values at the capillary scale^39^.

Mechano-pathological features of tumors have a crucial role in metastasis, invasion, and growth^47^. IFP is a metric of wide clinical significance, especially in chemotherapy and immunotherapy^12^. According to Fig. 5a, generally in all examined networks, the highest value of IFP is seen in tumor tissue because of the lack of an efficient lymphatic system in the tumor area as well as the greater permeability of tumor vasculatures^14,37,73^. The average maximum IFP within the tumor regions is approximately in good agreement with those in numerical studies of Boucher et al.^48^, and Soltani et al.^7^, as well as experimental results of Huber et al.^49^ and Boucher and Jain^50^, which investigate the IFP in a range of 587 and 4200 Pa. Increasing IFP with tumor size is also reported in several experiments^48,51,52^ and human tumors^53,54^. Since the IFV value is only proportional to the IFP gradient, according to Darcy's law, and the IFP gradient has a uniform distribution in tumor tissue, the IFV has very low values throughout the tumor central areas^12,55–57^. The maximum IFV occurs near the tumor border, where the IFP drop is precipitous, which is exactly matched to the measured values in the previous numerical and experimental studies^12,55,57,58,73^. Such very low IFV seen in the present results is also completely consistent with the more recent in vivo study^47^. The negligible impact of the convection term in CDR equations on FDG transport (the first term in Eq. 5) is related to these low IFV values, as reported by Soltani et al.^7^.

Detecting the tumor tissue by intracellular FDG concentration to analyze cancer staging, restaging, treatment response evaluation, or even predicting the risk of some diseases has been raised as important clinical roles of PET imaging^2,41^. Based on the spatiotemporal distribution of different FDG concentrations (see Supplementary Fig. S3), in all the snapshots, elevated FDG uptake can be seen in the tumor compared to surrounding healthy tissue, which is referred to as several biological and physiological features. Such radiopharmaceutical distribution within TME, as seen in Fig. 6, qualitatively corresponds to the outputs of the PET imaging device. Higher permeability of tumor-associated microvasculature as well as greater MVD within the tumor result in much larger extravasation of FDG into the TME. In addition, the FDG consumption of tumor cells is higher than healthy cells, due to their greater energy demand for cellular processes.

Spatiotemporal distribution of total concentration (see Supplementary Fig. S3d and Fig. 6) shows that at each time post-injection, by increasing the tumor size, concentrations within a tumor region are changed into a more homogenous structure. The capillary network architectures in each stage of tumor growth clearly affect the FDG uptake and its distribution within the tumor. Higher absorption of FDG by larger tumors implies that more detailed description of tumor physiology can be extracted in larger tumors compared to the smaller ones which are in the initial stages of tumor angiogenesis. Since the microvascular networks act as a source term for extravasation of radiopharmaceuticals to the interstitium (see Fig. 1), increasing the MVD in larger tumors leads to an increase in average total FDG uptake by tumors. This relation with source terms related to flow behavior and radiopharmaceutical concentration can be concluded mathematically by the two last terms in Eq. (5), which are related to the fluid flow behavior. Furthermore, increasing the MVD in larger tumors and more heterogeneous distribution of microvascular networks throughout larger tumor regions induce the extravasated radiopharmaceutical to have better transport within tumor tissue. In other words, by increasing MVD, a greater proportion of tumor surfaces can be covered in larger tumors. This phenomenon can also be explained by the two first terms in Eq. (5), which are related to the convection and diffusion transport mechanisms of FDG. As reported by Soltani et al.^7^, the FDG transport via vascular diffusion mechanism in microvascular network with a greater MVD is about 30% higher than that with lower MVD.

In routine clinical examinations, decision-making is based on the visual or semi-quantitative assessment of spatial uptake patterns, where both the relative pattern and strength of uptake provide are of value to clinical tasks. Tumor regions in the explored networks are clearly distinguished from surrounded healthy tissues, exhibiting higher SUVs. Regardless of the tumor stage and post-injection time, in regions where the MVD is relatively lower, SUVs in the tumor areas exhibited relatively lower magnitudes. Additionally, since the amount of total concentration within the tumor tissue increases over time, the SUVs in all the examined networks also increase. Overall, larger solid tumors depict greater SUVs at all times, as a result of increases in MVDs in larger tumors. In other words, larger tumors with higher SUVs imply potentially hazardous microvascular networks in their mature phase. This issue is also noted in several recent in vivo studies^42,59,60^. In addition, the results of Fig. 8 demonstrate the measured SUVs for soft and tumor tissues to be in remarkable agreement with Al-Nabhani et al.^61^, which were in the range of 0.7 ± 0.3 and 1.9 ± 1.4, respectively.

Current quantitative and semi-quantitative analyses based on the spatiotemporal model of FDG uptake are also designed to examine the effect of the heterogeneous structure of tumor microvessels and tumor progression. As shown in Fig. 9a, SUV_mean_ increases over time during tumor progression, in which larger tumors (with everything else being the same) can depict higher uptakes. Spatiotemporal distribution of SUV and SUV-based parameters (e.g., SUV_mean_) demonstrate that MVD and tumor size play critical roles in the SUV distribution and its values, as reported in the literature^42^. Therefore, tumor progression, which may cause a rise in MVD, not only results in more uniform distribution of SUV, but also leads to relatively higher SUV_mean_. It should be noted that the stage of tumor progression (i.e., tumor size) and distribution of tumor-induced angiogenesis through the ECM (i.e., MVD) are inherently interdependent, in which the MVDs can be calculated by novel computerized image analysis within tumor areas and adjacent healthy tissues^62^, although such applications in routine clinical imaging will require advanced imaging (e.g., perfusion CT) and inverse problems^7,19,63^ which are topics of future research. These findings suggest that correction of SUV uptake based on tumor size and MVD may better reflect accurate tumor glucose utilization levels for clinical assessments. These consequences are consistent with the results of an in vivo study^42^, which showed that adjustment of the SUV formula to consider the effect of tumor size should be applied. In addition, the values of the present SUVs and their trend have remarkable agreements with the recent experimental studies^64–66^.

Several factors can impact the resolution and accuracy of PET images, which may ultimately influence clinical interpretations. For instance, involuntary motions may affect the detection of microenvironment details during PET imaging. In particular, respiratory movements can result in displacements as large as 2 cm (e.g., lung nodules in the lower lobes or aorta^67,68^). This may cause the heterogeneity observed in our simulation results to be undetectable or significantly reduced in routine PET imaging. In addition, when existing scan times are in the order of minutes (thus adding possible voluntary movements by patients) this issue can be further amplified. Furthermore, there are a number of resolution degrading phenomena (e.g., inter-crystal blurring) that can degrade qualify of PET images (even in the absence of motion), culminating in partial volume effects^69,70^. In any case, the developed heterogeneity models, such as in this work, have value in understanding the biophysics of microenvironments. Furthermore, with extensive, ongoing research towards higher-resolution, significantly-less-degraded PET images (e.g., much shorter scans using high-sensitivity, long axial field-of-view PET scanners, data-driven motion correction methods, and higher resolution PET scanners), it is expected that greater heterogeneity of radiopharmaceutical uptakes can be quantified in next-generation PET imaging.

### Clinical relevance and perspective works

Results of the present study reflect how mathematical modeling and computational tools can be developed synergistically to accurately reproduce synthetic images of PET scan. The importance of such an approach to cancer research and clinical translation cannot be underestimated. Guaranteeing that computational simulations are accurate and valid is a crucial stage to bridge the gap between clinicians and technical researchers. Results of the present work, considering the formation of new capillaries by complex tumor angiogenesis process, interstitial and intravascular fluid flow, the average concentration of FDG uptake by tumors, as well as the SUV-related parameters, have been accurately verified through quantitative and/or qualitative interpretations. However, in order to validate the heterogeneous distribution of the radiopharmaceutical at the micrometer scale, a microscopic approach with optical/radioisotope features such as the combination of phase contrast microscopy^71^ with microscopic autoradiography^72^ is needed in our future works, which allows detection of uptake gradients in the background of the microvasculature. Once the outcomes are validated, such computational models can be employed to produce synthetic images of PET scan imaging at different stages of tumor growth with highly complicated radiotracer distribution and morphological details within the TME that would be difficult to investigate and visualize by PET scan technique.

Such a spatiotemporal model can be used to create a comprehensive atlas of FDG-based features (or any other radiopharmaceuticals) in various sizes of solid tumors. Using these snapshots of FDG uptake, presented in the proposed atlas, the optimum times for taking a series of images, recorded by a camera during PET imaging in each size of the tumor, can be predicted. These reference images can also be employed as an inverse engineering problem to estimate vital biological parameters of interest, for instance, MVD, diffusion coefficient, and kinetic parameters (${K}_{i}$), which play important role in designing new patient-specific drugs for personalized medicine. Additionally, applying novel artificial intelligence (AI) techniques (e.g., generative adversarial networks) to a larger dataset of such series of snapshots can generate massive synthesized PET images to solve the problem posed by the small medical samples in the conventional deep learning models^73^.

Radiation exposure of patients undergoing nuclear medicine imaging is a common concern. Low-dose PET images are noisy and have low quality. Another important aspect of the present model is its potential for hybridization with modern AI approaches to estimate and produce full-dose PET images extracted from low-dose PET images using intended image collection to assist clinicians and radiologists in their procedural planning. In addition, using this technique not only can result in much lower imaging doses compared to conventional doses, but may also assist to minimize the time duration of FDG-PET imaging, especially in whole-body PET imaging.

## Conclusion

A multi-scale computational model is presented and utilized to examine the effect of different stages of tumor growth and angiogenesis on biological features of interstitial and intravascular flow, as well as routine clinical radiotracer metrics used for nuclear medicine imaging. The mathematical model includes CDR equations to accurately investigate the spatiotemporal distribution of the FDG radiopharmaceutical by taking into account transport through diffusion and convection mechanisms from microvessel to tissue or within the tissue. Eventually, intravascular pressure, IFV, IFP, and various FDG concentrations—including extracellular, intracellular, phosphorylated—SUV-based metrics and related TACs are calculated based on real physiological data and biological considerations. The major findings of the study are:

- As the tumor grows, the IFP values also increase.
- Due to the higher permeability of tumor microvasculature as well as the lack of an efficient lymphatic system in the tumor region, in all examined vascularized tumors, the tumor tissue can be distinguished by higher FDG uptake compared to surrounded healthy tissues.
- Spatiotemporal distribution of FDG during each phase of tumor angiogenesis is greatly affected by the architecture of the microvessels and tumor stages.
- In the initial stage of tumor growth, radiotracer transport into the cancerous cells is limited due to the lower MVD.
- In larger tumors, due to the increase in MVD, the distribution of FDG changes to be more homogeneous within the tumor tissue.
- SUVs have relatively smaller magnitudes in tumor areas with poor MVD.
- SUV_mean_ in all examined tumor sizes increases over time, while larger tumor sizes with higher MVD have higher SUV_mean_ values.
- Tumor size and MVD can be integrated with SUV computations to enable more meaningful metrics.

The developed model can be utilized to generate different series of synthetic images for clinically relevant biological markers. Moreover, this framework can also be used to estimate the biological characteristics in each patient-specific microvascular network. Ultimately, this study is presented to improve understanding of FDG dynamics and to help bridge the gap between technical researchers, radiologists, and clinicians as a step forward toward personalized medicine.

## Supplementary Information

Supplementary Information.

Supplementary Video 1.

Supplementary Video 2.

## Data Availability

All data used for this study are available from the author upon request.

## Abbreviations

PET: Positron emission tomography
FDG: F-18 fluorodeoxyglucose
ECM: Extracellular matrix
GLUT: Glucose transporter
ROI: Region of interest
SDM: Spatiotemporal distribution model
PDE: Partial differential equation
ODE: Ordinary differential equation
TME: Tumor microenvironment
MVD: Microvascular density
VEGF: Vascular endothelial growth factor
EC: Endothelial cell
SUV: Standardized uptake value
TAC: Time-activity curve
IFV: Interstitial fluid velocity
IFP: Interstitial fluid pressure
CDR: Convection–diffusion–reaction
FDG-6-P: Phosphorylated intracellular FDG
CFD: Computational fluid dynamics
AI: Artificial intelligence
