# Supplementary material for: A spatiotemporal multi-scale computational model for FDG PET imaging at different stages of tumor growth and angiogenesis
Source: Sci Rep. 2022 Jun 16;12:10062. doi: 10.1038/s41598-022-13345-4 (PMC9203789; doi:10.1038/s41598-022-13345-4)
Supplement: Supplementary file 1 — Supplementary Information. [file 41598_2022_13345_MOESM1_ESM.docx]

**Supplementary File**

**A Spatiotemporal Multi-Scale Computational Model for FDG PET Imaging at Different Stages of Tumor Growth and Angiogenesis**

Farshad Moradi Kashkooli^1,2,†^, Mohammad Amin Abazari^1,†^, M. Soltani^1,3,4,5,*^, Mehran Akbarpour Ghazani^1,6^, and Arman Rahmim^7,8,9^

^1^ Department of Mechanical Engineering, K. N. Toosi University of Technology, Tehran, Iran

^2^ Department of Physics, Ryerson University, Toronto, ON, Canada

^3^ Department of Electrical and Computer Engineering, Faculty of Engineering, School of Optometry and Vision Science, Faculty of Science, University of Waterloo, Waterloo, Canada

^4^ Advanced Bioengineering Initiative Center, Multidisciplinary International Complex, K. N. Toosi University of Technology, Tehran, Iran

^5^ Centre for Biotechnology and Bioengineering (CBB), University of Waterloo, Waterloo, ON, Canada

^6^ Faculty of Mechanical Engineering, University of Tabriz, Tabriz, Iran

^7^ Department of Integrative Oncology, BC Cancer Research Institute, Vancouver, BC, Canada

^8^ Department of Physics & Astronomy, University of British Columbia, Vancouver, BC, Canada

^9^ Department of Radiology, University of British Columbia, Vancouver, BC, Canada

^*^Corresponding author: Email: msoltani@uwaterloo.ca

^†^ Equal contributions.

Supplementary File:

- Angiogenesis modeling, hemodynamics and intravascular fluid flow
- Hemorheology and structural adaptation
- Numerical Method
- Initial conditions
- References
- Supplemental Figures – S1 to S4

**Angiogenesis modeling**

Growth of a solid tumor is limited by a certain size of 2-3 mm without angiogenesis, the process of formation of neovasculature from pre-existing vessels [1]. This seminal phenomenon in tumor microenvironment has been studied numerically and experimentally from its first introduction by Judah Folkman as a governing factor in solid tumor growth [2]. It can be concluded that any model related to study of tumor microenvironment, requires containing angiogenesis modeling. To come closer to this end, a discrete probabilistic angiogenesis model proposed by Anderson et al. [3] and modified by Soltani et al. [4] has been used to generate the microvascular network. In this model, sprout formation, vessel movement, branching, anastomosis, blood flow rate, and hematocrit have been obtained.

It is observed that cells move in the domain based on three mechanisms: (1) random movement, which is interpreted as diffusion in the formulations; (2) chemotaxis, which is direct movement of vessels due to proangiogenic agent’s gradient; and (3) haptotaxis, which is transverse movement of vessels as a consequence of fibronectin presence in the tissue. The flux of endothelial cells (ECs) can be formulated as below:

| $J_{n}=J_{\text{random}}+J_{\text{chemo}}+J_{\text{hapto}}$ | (1) |
| --- | --- |

where $J_{n}$ is the total flux of ECs, $J_{\mathrm{rand}}$ is the flux obtained by random movement of ECs as diffusion in the domain, $J_{\mathrm{chemo}}$ is chemotactic movement of ECs, and $J_{\mathrm{hapto}}$ is the flux associated to haptotactic movement. By replacing the flux terms with corresponding definitions (references [3, 4]), Eq. 2 is reached as:

| $\frac{\partial n}{\partial t}=D_{n}\nabla^{2}n-R(\rho)\nabla.\left( \chi\left( c \right)n\nabla c \right)-\nabla.\left( \rho_{0}n\nabla f \right)$ | (2) |
| --- | --- |

In Eq. 2, EC density is represented by n, ECs coefficient of diffusion is *D_n_*, chemotactic function is *χ(c),* $\rho_{0}$ is a constant coefficient and *c*, *f* are concentrations of vascular endothelial growth factor (VEGF) and fibronectin, respectively. Also, $R(\rho)$ represents matrix density function.

ECs produce and secrete many matrix degrading enzymes (MDEs). Hence, a chemical agent is chosen to be representative of MDEs in the model. The equation for MDE that is presented by McDougall et al. [5] is modified and presented as:

| $\frac{\partial m}{\partial t}=\gamma n+\varepsilon\nabla^{2}m-\upsilon m$ | (3) |
| --- | --- |

Fibronectin is an omnipresent extracellular matrix (ECM) glycoprotein which plays a critical role in cell adhesion and movement of ECs [6]. Fibronectin, which is bound to ECM, assists the cells to perform transversal movements in the domain. Fibronectin is a ubiquitous chemical in the mammalian tissues. It is secreted and consumed by ECs, as they move in the domain:

| $\frac{\partial f}{\partial t}=\omega n-\xi mf$ | (4) |
| --- | --- |

where *ω* and *µ* are positive constants. Fibronectin is secreted by ECs, hence it is included in the first term and is degraded by MDEs which is implied in the second term of Eq. 4.

Hypoxic tumor cells secrete VEGF which is transported in the domain by diffusion. Moreover, as ECs move in the domain at the direction of VEGF gradient, VEGF is taken up by the ECs. In the absence of presence of tumor cells in the model, the model of angiogenesis includes VEGF consumption by ECs as below:

| $\frac{\partial c}{\partial t}=-\lambda nc$ | (5) |
| --- | --- |

in which *λ* is a dimensionless constant.

Reference variables of $c_{0},\text{ }f_{0},\text{ }n_{0},\text{ and }\tau$ are used to non-dimensionalize main parameters of the equations which are shown below:

$$\tilde{c}=\frac{c}{c_{0}},\text{ }\tilde{f}=\frac{f}{f_{0}},\text{ }\tilde{n}=\frac{n}{n_{0}},\text{ }\tilde{t}\text{ =}\frac{t}{\tau}$$

τ is defined as $\frac{L^{2}}{D_{c}}$ where *L* is length scale in the simulations which shows the tumor-parent vessel distance. τ is dependent upon *L* and is interpreted in days.

By application of the above parameters to the equations, non-dimensional form of equations are reached as:

| $\frac{\partial n}{\partial t}=D\nabla^{2}n-Z(\rho)\nabla.[\chi(1+\delta c)n\nabla c+\varphi n\nabla f]$ | (6) |
| --- | --- |
| $\frac{\partial c}{\partial t}=-\eta nc$ |  |
| $\frac{\partial f}{\partial t}=\beta n-\zeta mf$ |  |
| $\frac{\partial m}{\partial t}=\alpha n+\varepsilon\nabla^{2}m-\nu m$ |  |

where $Z(\rho)$ is the non-dimensionalized form of the matrix density function as in equation (7) and *D*, *χ*, δ, ϕ, *η*, *β*, *ζ, α, ε, and υ* are coefficients which have been non-dimensionalized and are positive. The aforementioned parameters are clearly introduced and obtained in [3, 4].

| $Z(\rho)=exp[-\frac{{(\rho-\rho_{0})}^{2}}{2\sigma^{2}}]$ | (7) |
| --- | --- |

where *ρ*, $\rho_{0}$, and σ are matrix density, reference matrix density and a constant in the equation. The parameters in equations (6) and (7) are obtained from references [3, 4]. Tilde, (⁓), has been omitted from the above equations due to brevity.

Euler finite difference method is utilized to discretize the equations. After discretization, a set of formulations has been reached that is interpreted by probabilistic definitions. The movement of an EC has been determined by generation of a random number and based on the discretized formulations. Interested reader may refer to [3, 4].

ECs move towards the tumor as filopodia route the way by sensing gradients of chemical agents. ECs branch as they get closer to the tumor. Branching conditions are divided into three parts: (1) the vessel is mature enough based on its age, (18 hours); (2) density of ECs is enough for branching; (3) there is free space for the daughter cell. In addition to these conditions, a probability function has been considered to determine the branching occurrence at a point as below:

| $\left\{ \begin{aligned} P_{\text{branching}}(x,y)=0,\text{ }c(x,y)\leq0.5 \\ P_{\text{branching}}(x,y)=0.3,\text{ }0.5<c(x,y)\leq0.65 \\ P_{\text{branching}}(x,y)=0.5,\text{ }0.65<c(x,y)\leq0.8 \\ P_{\text{branching}}(x,y)=1,\text{ }c(x,y)>0.8 \end{aligned} \right.$ | (8) |
| --- | --- |

Prior to angiogenesis onset, sprouts appear on the parent vessels. VEGF as the main tumor angiogenic factor (TAF) in the domain is the propellant of this phenomenon. If VEGF concentration on a parent vessel reaches a threshold value, the vessel starts to form new sprouts. As a new sprout protrudes out of a vessel at a point, signals are sent at a limited distance to prevent nearby stalk cells to turn into new sprouts. The location of these sprouts are not only governed by biological conditions, but also probability is utilized as a part of this process. In this research, the modality used by Secomb et al. [7] is adopted to predict the number of initial sprouts and corresponding locations. VEGF concentration is the main factor controlling the behavior of sprouts defined as below:

| $P_{\text{sprout}}=\left\{ \begin{aligned} k_{p}l_{\text{seg}}\text{Δ}t\left( \frac{c\left( i,j \right)-c_{\text{th}}}{c\left( i,j \right)-c_{\text{th}}+c_{\text{th,50}}} \right)\text{ }\text{ }\text{ if }c\left( i,j \right)>c_{\text{th}} \\ 0\text{ }\text{ }\text{ if } c\left( i,j \right)<c_{\text{th}} \end{aligned} \right.$ | (9) |
| --- | --- |

where *k*_p_ is maximum probability of branching per parent vessel length and time, *l*_seg_ is the length of the nominated parent vessel, Δ*t* is simulation time step and *c*_th_ and *c*_th, 50_ are positive threshold values. Corrsponding values for parameters are tabulated in Table 1.

**Table 1**. Parameters of initial sprouting.

| Parameter | Value | Dimension | Reference |
| --- | --- | --- | --- |
| $k_{\text{P}}$ | $2\times10^{-3}$ | $\frac{\text{1}}{\text{μm.day}}$ | [7] |
| $l_{\text{seg}}$ | 5 | cm | - |
| $c_{\text{th}}$ |  | M | [7] |
| $c_{\text{th,50}}$ |  | M | [7] |

* These parameters have been changed to fit this model.

ECs move in the domain and encounter each other. The filopodia existing at the tip of the vessel perfuse the other ECs and make a connection. Hence, closed loops which blood can flow is created. Blood as a biphasic fluid contains erythrocytes, leukocytes, platelets and plasma flowing in the vasculature. The flow velocity is such that flow regime is laminar in the microvessels. Accordingly, Hagen-Poiseuille’s law governs the flow as below:

| $Q_{\text{v}}=\frac{\text{π}D_{\text{v}}^{4}}{128L_{\text{v}}}\frac{\Delta P_{\text{B}}}{\mu_{\text{app}}\left( D_{\text{v}},\text{ }H_{D} \right)}$ | (10) |
| --- | --- |

where *∆P_B_* is pressure difference for a microvessel segment, *D*_v_ is the diameter of the microvessel, *L*_v_ is the microvessel length. Apparent blood viscosity, *µ*_app_ (*D*_v_*, H_D_*), is a function of microvessel diameter and hematocrit.

Red blood cell distribution, which is normally about 0.45 of total blood volume, known as hematocrit distribution, are different at bifurcations. It is observed that as blood is divided into two outlets at a bifurcation in a microvascular network, blood hematocrit will be other than 0.45 in the daughter vessels. For the case of blood entering a junction by two vessels and discharging to an outlet, the blood flow rate and erythrocyte flow rate can easily be obtained by the sum of two inlet flows. On the other hand, if blood flow enters a junction by a mother vessel and discharges into two daughter vessels, the corresponding blood flow rates and erythrocyte flow rates can be obtained by the following equation:

| $\left\{ \begin{aligned} \text{FQ}_{\text{E}}=0\text{ }\text{ }\text{ if }\text{FQ}_{\text{B}}\text{< }\text{X}_{0} \\ \log\text{it FQ}_{\text{E}}=A+B\log\text{it}\left[ \left( \text{FQ}_{\text{B}}-\text{X}_{0} \right)/\left( 1-2\text{X}_{\text{0}} \right) \right]\text{ }\text{ }\text{ if }\text{X}_{\text{0}}\text{< }\text{FQ}_{\text{B}}\text{<1-X}_{\text{0}} \\ \text{FQ}_{E}=1\text{ if }\text{FQ}_{\text{B}}\text{ > }{\text{1}\text{-X}}_{\text{0}} \end{aligned} \right.$ | (11) |
| --- | --- |

where logit *x* = ln [(*x*/(1-*x*))] and *A, B* and *X_0_* are defined below:

| $A=-13.29\left[ \frac{{D_{\text{α}}^{2}}/{D_{\text{β}}^{2}-1}}{{D_{\text{α}}^{2}}/{D_{\text{β}}^{2}+1}} \right]\frac{\left( 1-H_{D} \right)}{D_{\text{F}}}$ | (12) |
| --- | --- |
| $B=1+6.98\frac{\left( 1-H_{D} \right)}{D_{\text{F}}}$ | (13) |
| $X_{0}=0.964\frac{\left( 1-H_{D} \right)}{D_{\text{F}}}$ | (14) |

Where *D*_α_ and *D*_β_ are diameters of daughter microvessels and the diameter of inlet microvessel to the nominated junction is shown by *D*_F_. *Q*_F_ and *H_D,_*_F_ are blood and erythrocyte flow rates of the inlet vessel to the junction, while *H_D,2_* and *H_D,3_,* are hematocrits of daughter branches, and ${FQ}_{E}=\frac{\tilde{Q}_{a}}{\tilde{Q}_{F}}$and ${FQ}_{B}=\frac{Q_{a}}{Q_{F}}$are volumetric flow rate of red blood cells and blood cells of a daughter vessel to corresponding values of the mother vessel at the junction, respectively. Tilde, (~), is inserted to show erythrocyte flow rate.

**Blood viscosity and structural adaptation**

Blood contains different constituents which makes its properties variable based on the concentration of the compositions. One of the most important constituents influencing the blood viscosity is the fraction of red blood cells. Apparent blood viscosity is a function of both microvessel hematocrit and its diameter as described by Pries eta al. [8].

| $\mu_{\text{app}}\left( D,\text{ }H_{D} \right)=\mu_{\text{rel}}\times\mu_{\text{plasma}}$ | (15) |
| --- | --- |

In which *µ*_plasma_ is a constant value of 1.2 cp representing plasma viscosity. *µ*_rel_ is defined as equation (16):

| $\mu_{\text{rel}}\left( D,H_{D} \right)=\left[ 1+\left( \mu_{0.45}-1 \right)f\left( H_{D} \right)\left( \frac{D_{\text{v}}}{D_{\text{v}}-1.1} \right)^{2} \right]\left( \frac{D_{\text{v}}}{D_{\text{v}}-1.1} \right)^{2}$ | (16) |
| --- | --- |

where *µ*_0.45_ is the viscosity when red blood cell distribution is normal in the microvessel and *D*_v_ is its diameter. Function *f* (*H_D_*) represents the dependency of blood viscosity to hematocrit. The equations for the aforementioned terms are as follows:

| $\mu_{0.45}=6e^{-0.085D_{\text{v}}}+3.2-2.44e^{-0.06\left( D_{\text{v}} \right)^{0.0645}}$ | (17) |
| --- | --- |
| $f\left( H_{D} \right)=\frac{\left( 1-H_{D} \right)^{C}-1}{\left( 1-0.45 \right)^{C}-1}$ | (18) |
| $C=\left( 0.8+e^{-0.075D_{\text{v}}} \right)\left( -1+\frac{1}{1+{10}^{-11}\left( D_{\text{v}} \right)^{12}} \right)+\left( \frac{1}{1+{10}^{-11}\left( D_{\text{v}} \right)^{12}} \right)$ | (19) |

Vessel walls experience shear stress as blood flows in the vascular network. Moreover, metabolic stimuli affect the blood flow rate which consequently leads to reconstruction of microvasculature. Pries et al. [9] have experimentally studied this phenomenon and suggested the related formulations. The rate of change of vessel diameter is dependent upon three different stimuli which is interpreted as the total stimulus in the equation below:

| $\text{Δ}D_{\text{v}}=S_{\text{tot}}D_{\text{v}}\text{Δ}t$ | (20) |
| --- | --- |

where diameter variance Δ*D*_v_ is amount change in microvessel diameter with an initial vessel diameter *D*_v_ in a time step Δ*t*. Summing wall shear stimulus, *S*_wss_, vessel transmural pressure stimulus, *S*_p_, metabolic stimulus, *S*_m_, total stimulus is reached as:

| $S_{\text{tot}}=S_{\text{wss}}+S_{\text{p}}+S_{\text{m}}$ | (21) |
| --- | --- |

Wall shear stress stimulus is a logarithmic function of wall shear stress, τ_w_:

| $S_{wss}=\text{log}\left( \tau_{w}+\tau_{ref} \right)$ | (22) |
| --- | --- |

| $\tau_{w}=\frac{32\mu_{\text{app}}\left( D_{\text{v}},H_{D} \right)}{\pi D_{\text{v}}^{3}}\left\vert Q_{\text{v}} \right\vert$ | (23) |
| --- | --- |

Transmural pressure also contributes to total stimulus as shown in equations (24) and (25).

| $\tau_{e}\left( P_{\text{V}} \right)=100-86\text{ }\exp\left[ -5000.\left[ \log\left( \text{log}P_{\text{V}} \right) \right]^{5.4} \right]$ | (24) |
| --- | --- |

| $S_{p}=-\text{log}\tau_{e}\left( P \right)$ | (25) |
| --- | --- |

Metabolic conditions in the tumor microenvironment affects reconstruction of the vasculature as metabolic stimulus shown below:

| $S_{\text{m}}=k_{\text{m}}\text{log}\left( \frac{Q_{\text{ref}}}{QH_{D}}+1 \right)$ | (26) |
| --- | --- |

where *k*_m_ is a positive constant and *Q*_ref_ is the blood flow rate in the parent vessel.

The rate of change in the diameter of a microvascular segment is dependent on all the previously defined stimuli which is represented in Eq. 27:

| $\text{Δ}D_{\text{v}}=\left[ \text{log}\left( \tau_{w}+\tau_{\text{ref}} \right)-k_{p}\text{log}\tau_{e}\left( P_{\text{V}} \right)+k_{m}\log\left( \frac{Q_{\text{ref}}}{QH_{D}}+1 \right)-k_{s} \right]D_{\text{v}}\text{Δ}t_{\text{d}}$ | (27) |
| --- | --- |

in which *k*_s_ is the shrinking tendency of vessels.

Since a microvascular network is composed of many microvascular segments, a change in the diameter of a single vessel affects the blood flow in the other vessel segments. Therefore, the process of network reconstruction continues until an equilibrium is reached.

At the time the equilibrium has been reached in the network, some of the vessel segments are prone to form new sprouts based on the structural and environmental conditions. Microvessels which experience a high wall shear stress and are exposed to high concentrations of VEGF will branch. The probability of branching due to wall shear stress is the same as [10]. Probability of wall shear induced branching for a vascular segment is tabulated in Table 2.

**Table 2.** Vessel branching probability based on TAF concentration and wall shear stress (WSS).

| [WSS]  [TAF] | $\leq0.4$ | $0.4-0.6$ | $0.6-0.8$ | $0.8\leq$ |
| --- | --- | --- | --- | --- |
| $\leq0.1$ | 0 | 0 | 0 | 0 |
| $0.1-0.2$ | 0 | 0 | 0.1 | 0.3 |
| $0.2-0.3$ | 0 | 0.1 | 0.5 | 0.5 |
| $0.3-0.4$ | 0 | 0.4 | 0.8 | 0.6 |
| $0.4\leq$ | 0 | 0.7 | 1.0 | 1.0 |

**Numerical method**

The computational domain is an equilateral rectangular section of 5 cm each side. There are 500 × 500 lattice sites in the domain and the ECs are considered to be 10 µm. Therefore, each lattice can contain 100 ECs. It has been observed that ECs can move as ensembles of cells in a tissue [11]. This behavior has been supposed here to decrease time and cost of the simulations.

The non-dimensional time step has chosen in accordance to exhibit the physical movement of the ECs in a tissue as it is 14 µm/hr [12, 13].

Neumann boundary condition has been considered on domain boundaries for angiogenesis in the form of Eq. 28:

| $\zeta.\left[ D_{n}\nabla^{2}n-R(\rho)\nabla.\left( \chi\left( c \right)n\nabla c \right)-\nabla.\left( \rho_{0}n\nabla f \right) \right]=0$ | (28) |
| --- | --- |

where *ζ* is an outward unit normal vector.

**Initial conditions**

An initial concentration of VEGF is assumed as it has been secreted by a circular tumor located at the center of the domain [3]:

| $c\left( x,y,0 \right)=\left\{ \begin{aligned} 1,\text{ }0\leq r\leq R, \\ \frac{\left( \kappa-r \right)^{2}}{\kappa-0.1},\text{ }R<r\leq1 \end{aligned} \right.$ | (29) |
| --- | --- |
| $r=\sqrt{\left( x-\frac{1}{2} \right)^{2}+\left( y-\frac{1}{2} \right)^{2}}$ |  |

where *R* is the radius of the tumor and 𝜅 is a constant.

Since ECs are the main producers of MDE in the domain, initial distribution of MDE is in accordance with the initial EC density distribution which is a parent vessel in this simulation. For fibronectin initial concentration, a constant value of 0.5 is considered for all domain [4].

**Abbreviations:** EC: Endothelial cell; VEGF: Vascular endothelial growth factor; MDE: Matrix degrading enzyme; ECM: Extracellular matrix; TAF: Tumor angiogenic factor.

**References**

[1] I. Zuazo-Gaztelu and O. Casanovas, "Unraveling the role of angiogenesis in cancer ecosystems," *Frontiers in oncology,* vol. 8, p. 248, 2018.

[2] J. Folkman, "Tumor angiogenesis: therapeutic implications," *New england journal of medicine,* vol. 285, pp. 1182-1186, 1971.

[3] A. R. Anderson and M. Chaplain, "Continuous and discrete mathematical models of tumor-induced angiogenesis," *Bulletin of mathematical biology,* vol. 60, pp. 857-899, 1998.

[4] M. Soltani, "Numerical Modeling of Drug Delivery to Solid Tumor Microvasculature," Ph.D., Chemical Engineering, University of Waterloo, 2013.

[5] S. R. McDougall, A. R. Anderson, and M. A. Chaplain, "Mathematical modelling of dynamic adaptive tumour-induced angiogenesis: clinical implications and therapeutic targeting strategies," *Journal of theoretical biology,* vol. 241, pp. 564-589, 2006.

[6] L. Parisi, A. Toffoli, B. Ghezzi, B. Mozzoni, S. Lumetti, and G. M. Macaluso, "A glance on the role of fibronectin in controlling cell response at biomaterial interface," *Japanese dental science review,* vol. 56, pp. 50-55, 2020.

[7] T. W. Secomb, J. P. Alberding, R. Hsu, M. W. Dewhirst, and A. R. Pries, "Angiogenesis: an adaptive dynamic biological patterning problem," *PLoS computational biology,* vol. 9, p. e1002983, 2013.

[8] A. Pries, T. Secomb, and P. Gaehtgens, "Biophysical aspects of blood flow in the microvasculature," *Cardiovascular research,* vol. 32, pp. 654-667, 1996.

[9] A. Pries, B. Reglin, and T. Secomb, "Structural adaptation of microvascular networks: functional roles of adaptive responses," *American Journal of Physiology-Heart and Circulatory Physiology,* vol. 281, pp. H1015-H1025, 2001.

[10] M. Akbarpour Ghazani, Z. Nouri, M. Saghafian, and M. Soltani, "Mathematical modeling reveals how the density of initial tumor and its distance to parent vessels alter the growth trend of vascular tumors," *Microcirculation,* vol. 27, p. e12584, 2020.

[11] A. Haeger, K. Wolf, M. M. Zegers, and P. Friedl, "Collective cell migration: guidance principles and hierarchies," *Trends in cell biology,* vol. 25, pp. 556-566, 2015.

[12] J. B. Kearney, N. C. Kappas, C. Ellerstrom, F. W. DiPaola, and V. L. Bautch, "The VEGF receptor flt-1 (VEGFR-1) is a positive modulator of vascular sprout formation and branching morphogenesis," *Blood,* vol. 103, pp. 4527-4535, 2004.

[13] H. Salavati and M. Soltani, "The Impact of Endothelial Cells Proliferation in a Multiscale Realistic Reproduction of Angiogenesis," *Biochemical Engineering Journal,* 2018.

**
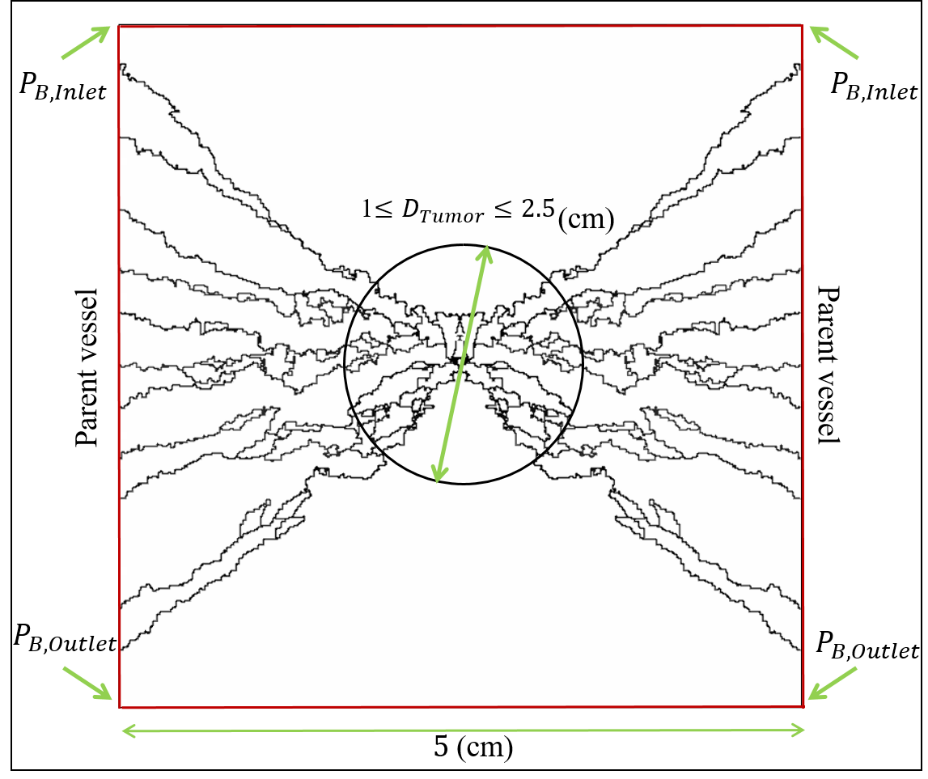
**

**Supplementary figure 1.** Schematic of computational domain consisted of solid tumor, healthy tissue, and capillary network. Boundary conditions used in intravascular blood flow simulation are also illustrated.


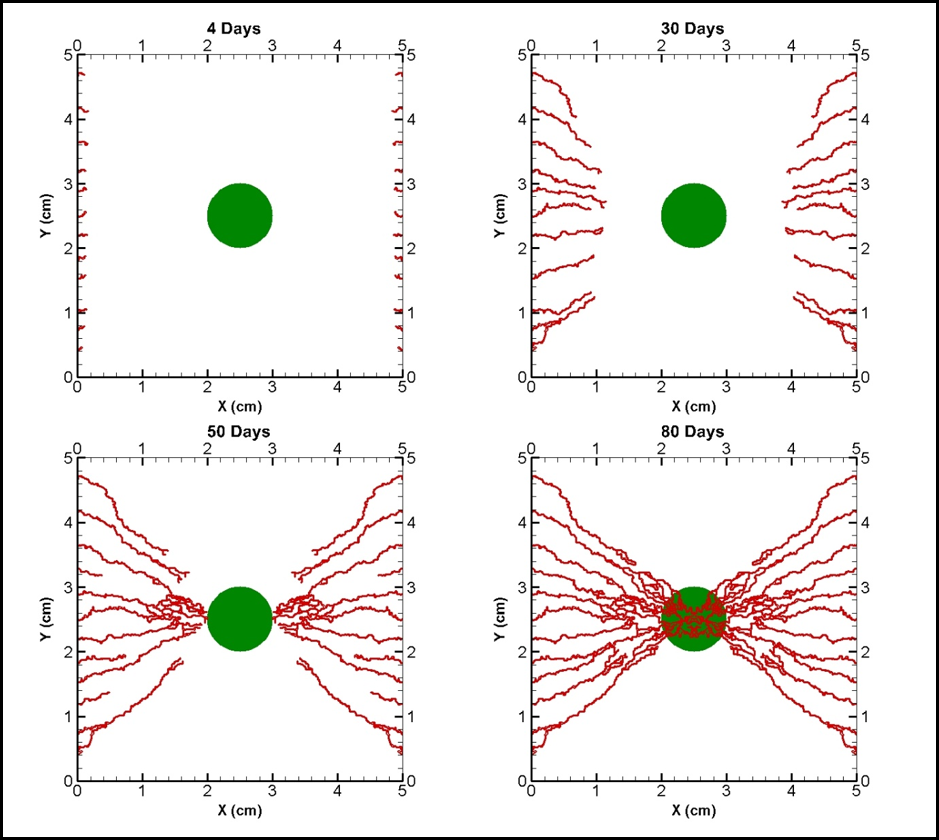


**Supplementary figure 2.** Construction of microvascular network in 1-cm tumor at days 4, 30, 50, and 80 of tumor-induced angiogenesis.


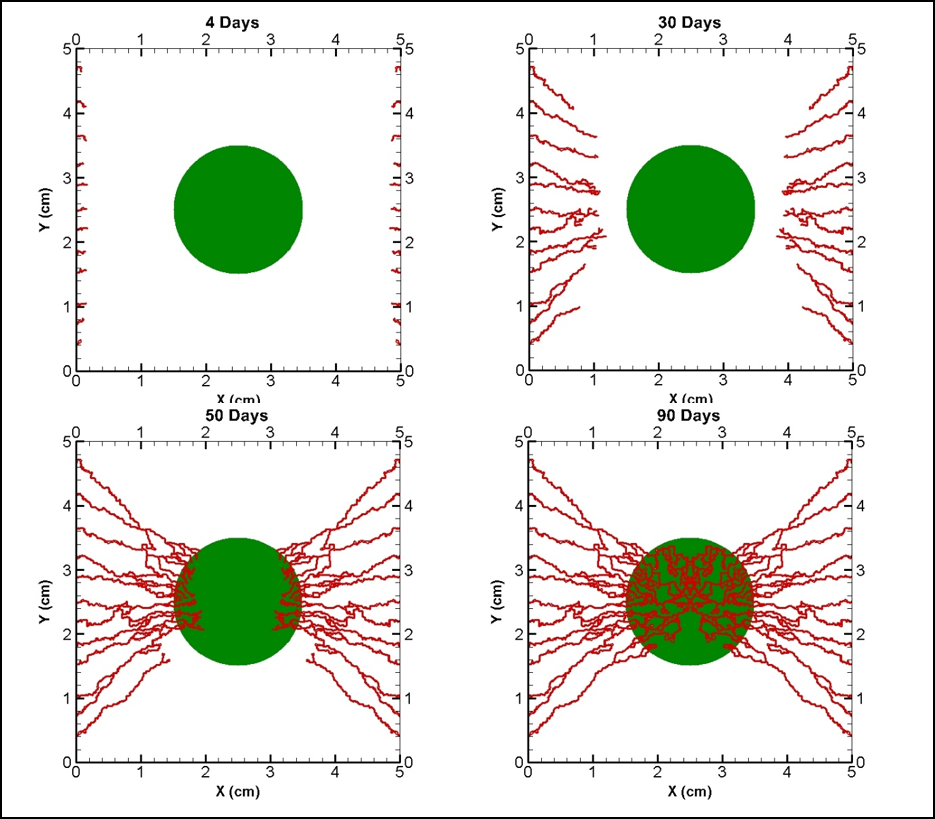


**Supplementary figure 3**. Construction of microvascular network in 2-cm tumor at days 4, 30, 50, and 90 of tumor-induced angiogenesis.

**
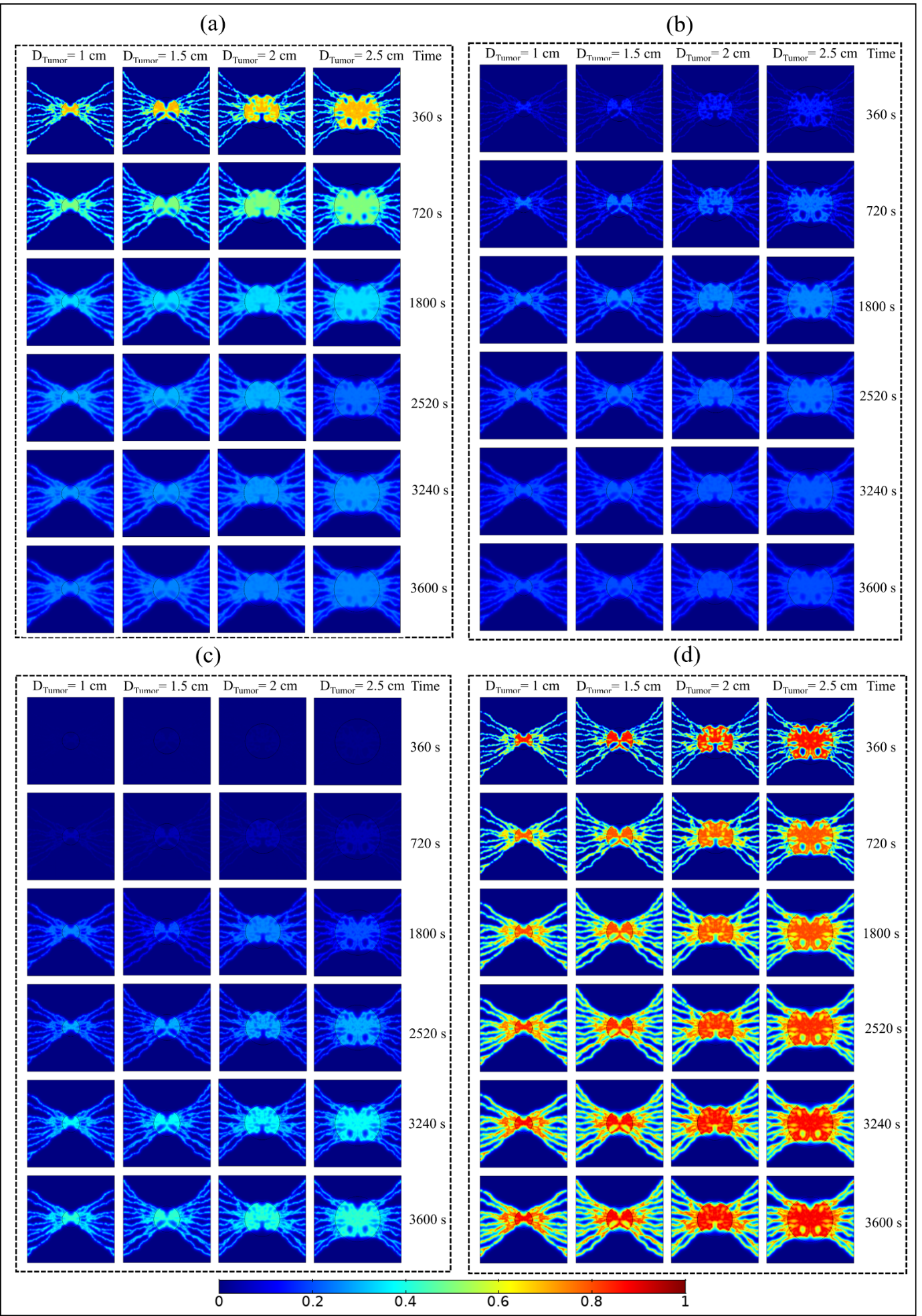
** **Supplementary figure 4.** Spatiotemporal distribution of non-dimensional **(a)** extracellular FDG concentration ($C_{i}$), **(b)** intracellular FDG concentration ($C_{e}$), **(c)** phosphorylated intracellular concentration ($C_{m}$), and **(d)** total FDG concentration ($C_{total}$) at different times.
